# Color-scalable flow cytometry with Raman tags

**DOI:** 10.1093/pnasnexus/pgad001

**Published:** 2023-01-14

**Authors:** Ryo Nishiyama, Kotaro Hiramatsu, Shintaro Kawamura, Kosuke Dodo, Kei Furuya, Julia Gala de Pablo, Shigekazu Takizawa, Wei Min, Mikiko Sodeoka, Keisuke Goda

**Affiliations:** Department of Chemistry, The University of Tokyo, Tokyo 113-0033, Japan; Department of Chemistry, The University of Tokyo, Tokyo 113-0033, Japan; Research Centre for Spectrochemistry, The University of Tokyo, Tokyo 113-0033, Japan; PRESTO, Japan Science and Technology Agency, Saitama 332-0012, Japan; Synthetic Organic Chemistry Laboratory, RIKEN Cluster for Pioneering Research, Saitama 351-0198, Japan; RIKEN Center for Sustainable Resource Science, Saitama 351-0198, Japan; Synthetic Organic Chemistry Laboratory, RIKEN Cluster for Pioneering Research, Saitama 351-0198, Japan; RIKEN Center for Sustainable Resource Science, Saitama 351-0198, Japan; Department of Chemistry, The University of Tokyo, Tokyo 113-0033, Japan; Department of Chemistry, The University of Tokyo, Tokyo 113-0033, Japan; Department of Chemistry, The University of Tokyo, Tokyo 113-0033, Japan; Department of Chemistry, Columbia University, New York , NY 10027, USA; Synthetic Organic Chemistry Laboratory, RIKEN Cluster for Pioneering Research, Saitama 351-0198, Japan; RIKEN Center for Sustainable Resource Science, Saitama 351-0198, Japan; Department of Chemistry, The University of Tokyo, Tokyo 113-0033, Japan; Department of Bioengineering, University of California, CA 90095, USA; Institute of Technological Sciences, Wuhan University, Wuhan 430072, China

**Keywords:** flow cytometry, Raman spectroscopy, Raman tag, Raman probe, endocytosis

## Abstract

Flow cytometry is an indispensable tool in biology and medicine for counting and analyzing cells in large heterogeneous populations. It identifies multiple characteristics of every single cell, typically via fluorescent probes that specifically bind to target molecules on the cell surface or within the cell. However, flow cytometry has a critical limitation: the color barrier. The number of chemical traits that can be simultaneously resolved is typically limited to several due to the spectral overlap between fluorescence signals from different fluorescent probes. Here, we present color-scalable flow cytometry based on coherent Raman flow cytometry with Raman tags to break the color barrier. This is made possible by combining a broadband Fourier-transform coherent anti-Stokes Raman scattering (FT-CARS) flow cytometer, resonance-enhanced cyanine-based Raman tags, and Raman-active dots (Rdots). Specifically, we synthesized 20 cyanine-based Raman tags whose Raman spectra are linearly independent in the fingerprint region (400 to 1,600 cm^−1^). For highly sensitive detection, we produced Rdots composed of 12 different Raman tags in polymer nanoparticles whose detection limit was as low as 12 nM for a short FT-CARS signal integration time of 420 µs. We performed multiplex flow cytometry of MCF-7 breast cancer cells stained by 12 different Rdots with a high classification accuracy of 98%. Moreover, we demonstrated a large-scale time-course analysis of endocytosis via the multiplex Raman flow cytometer. Our method can theoretically achieve flow cytometry of live cells with >140 colors based on a single excitation laser and a single detector without increasing instrument size, cost, or complexity.

Significance Statement The combination of high-speed Raman spectroscopy, Raman tags, and Raman-active dots enabled 12-color flow cytometry with high speed (up to 50 cells/s) and high accuracy of 98%. This method is extendable to >140 colors based on a single excitation laser and a single detector without increasing instrument size, cost, or complexity.

## Introduction

Flow cytometry is an indispensable tool in diverse areas of biology and medicine (e.g., cancer biology, immunology, microbiology, and COVID-19 biology) for counting and analyzing cells in large heterogeneous populations (1–5). In simple flow cytometry methods, rough morphological information such as cell diameter and granularity is obtained based on measurements of light scattering and electronic impedance (1). For chemical analysis, fluorescence detection is performed on each cell based on the use of fluorescent probes that specifically bind to target molecules on the cell surface or within the cell. Moreover, simultaneous analysis of target molecules is possible by using different types of fluorescent probes with different fluorescence emission wavelengths. This multiplexing technique is essential for maximizing the amount of information obtained in a single flow cytometry run (6, 7).

However, fluorescence flow cytometry has a critical limitation: the color barrier. The number of chemical traits that can be simultaneously resolved is typically limited to ∼10 due to the spectral overlap between fluorescence signals from different fluorescent probes (8–10). This limitation does not allow the study of highly heterogeneous cell populations such as immune cells. To break the color barrier, spectral flow cytometry has recently been proposed and demonstrated to simultaneously probe many molecules by its super-multiplex sensing capability (11–14). The emission spectrum of every fluorescent molecule is first detected with an optical spectrometer and is then followed by spectral unmixing to decode the combined fluorescence spectrum. However, fluorescence flow cytometry requires an increasing number of lasers to increase the number of colors, resulting in high complexity and cost. Another method that breaks the color barrier is mass cytometry based on labeling cells with heavy metal ion tags and time-of-flight mass spectrometry for measurements (15). Unfortunately, the destructive nature of mass cytometry (16) does not allow its application to temporal tracking or downstream analysis such as RNA sequencing and cultivation after cell sorting.

In this article, we present color-scalable flow cytometry based on coherent Raman flow cytometry with Raman tags to break the color barrier. Inspired by Raman tags for super-multiplex Raman imaging in the silent region (10, 17), this is made possible by combining (i) a high-throughput broadband Raman flow cytometer based on Fourier-transform coherent anti-Stokes Raman scattering (FT-CARS) (18–22), which employs a time-domain repetitive pump-probe scheme for rapid CARS measurements, (ii) resonance-enhanced cyanine-based Raman tags, and (iii) Raman-active dots (Rdots) (23, 24), which are nanoparticles (typically made of polymers) containing densely packed Raman tags. Specifically, to realize multiplex measurements, we developed a palette of 20 cyanine-based Raman tags (including eight newly synthesized cyanine molecules) whose Raman spectra are linearly independent in the fingerprint region (400 to 1,600 cm^−1^). To enhance the Raman signals from the developed tags, we produced 12 different Rdots composed of the cyanine-based Raman tags in polymer nanoparticles whose detection limit is as low as 12 nM for a short FT-CARS signal integration time of 420 µs. As a proof-of-concept demonstration, we performed multiplex flow cytometry of MCF-7 breast cancer cells stained by 12 different Rdots with a high classification accuracy of 98.0%. Moreover, to show a practical application of multiplex flow cytometry, we demonstrated large-scale time-course analysis of endocytosis by exposing cells to different Rdots at different timings and decoding their time-varying uptake of Rdots by spectral fitting. A major advantage of our multiplex flow cytometry with Raman tags over spectral flow cytometry and mass cytometry is that the number of molecules that can be simultaneously measured can theoretically scale up to 140 or even more with a single excitation laser and a single detector without the need for increasing the instrument size, cost, or complexity and without the disadvantage of destroying cells.

## Materials and methods

### Synthesis and characterization

Detailed synthesis (Schemes S1 to S10) and characterization data (Figs. S8 to S22) of all the compounds are shown in the Supplementary Information.

### Optical setup

A detailed schematic of our FT-CATS flow cytometer (18) is shown in Fig. S5. A Ti:sapphire laser (Coherent, Vitara, 780 nm, 17 fs) is used as a light source. For dispersion compensation, the output pulses pass through a pulse shaper and are reflected several times by a chirped mirror pair (DCMP175, Thorlabs). Then, the pulses are divided into pump-probe pulse pairs by a Michelson interferometer, with which pulse pairs with orthogonal polarizations are generated. In one of the interferometer arms, the optical path length is rapidly modulated by a resonant scanner (CRS 12 kHz, Cambridge Technology) formed in a 4-f optical system. An electronic synchronization signal generated by the resonant scanner is monitored at Ch. A of the digitizer (ATS9440, AlazarTech) in conjunction with the FT-CARS signal. The generated pulse pairs are focused onto the sample flowing in a lab-made microfluidic channel with an objective lens after passing through a long-pass filter (FELH0750, Thorlabs) to reduce the background signals. The FT-CARS signal generated at the sample is collected with another objective lens and detected by an avalanche photodiode (APD120A, Thorlabs). To isolate the blue-shifted FT-CARS signal from the incident light, the beam passes through a short-pass filter (FESH0750, Thorlabs) and a polarizer that is transmissive only for the probe polarization. The signal is then sampled at Ch. C of the digitizer at a sampling rate of 100 million samples/s. In flow cytometry measurements, data acquisition with the digitizer is triggered when Ch. C signal level exceeds a certain voltage. For each cell, 100,000 samples (1 ms), including 30,000 samples before the trigger position, are acquired. To calibrate the pump-probe delay, an interferogram generated by a continuous-wave laser (QLD1061, QD Laser, 1064 nm) is monitored at Ch. B. The CW beam is combined with the Ti:sapphire beam before entering the Michelson interferometer and separated from the Ti:sapphire beam after exiting the interferometer.

### Data analysis

Before data analysis, acquired FT-CARS signals are gated in the time domain. Some cells show sudden changes of the FT-CARS signal in the time domain (see Fig. S6) presumably due to laser-induced bubble generation in the cell, which significantly distorts the spectral profile after Fourier transformation. Therefore, data containing step-function-like behavior are excluded in the following analysis. After the gating, the single-cell trace containing 100,000 samples is divided into 22 to 23 traces corresponding to the single-scan data. In each trace, the optical delay of each FT-CARS signal is calibrated by using the CW interferogram acquired at Ch. B. Then, by Fourier-transforming the calibrated FT-CARS time-domain interferogram, a Raman spectrum is obtained. A single-cell Raman spectrum is obtained as an average of the 22 to 23 Raman spectra.

### Photostability of Raman tags

The photostability of the Raman tags was evaluated by measuring the temporal change of FT-CARS spectra of the IR740 solution (200 µM in methanol) under continuous exposure to incident light for 100 ms after opening a mechanical shutter (Newport 76,993) (Fig. S7). Their decay time constant was found to be about 6 ms, which was significantly longer than the duration of incident light exposure to each Raman tag (0.1 ms) in our flow cytometry measurements at a flow speed of 8 cm/s.

### Preparation of Rdots

**Fig. 1.**
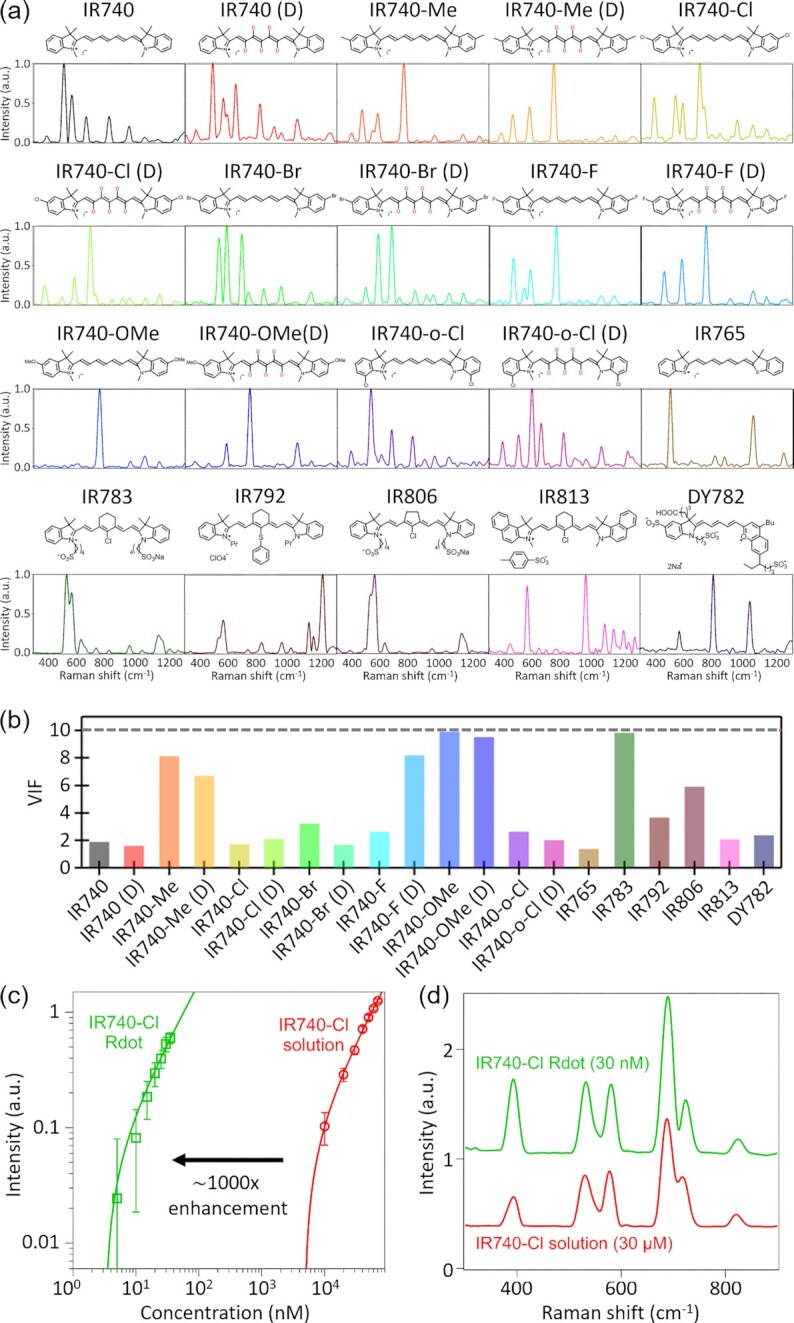
Characterization of cyanine-based Raman tags. (a) Molecular structures and Raman spectra of cyanine-based Raman tags. Each spectrum was obtained with our FT-CARS flow cytometer at a concentration of 100 µM and a measurement duration of 0.8 s. (b) VIF values of 20 cyanine-based Raman tags. (c) Concentration dependence of Raman intensities of IR740-Cl methanolic solutions and Rdot-IR740-Cl (measurement time: 0.42 ms). The error bars indicate the SDs of the measured FT-CARS intensities. (d) Raman spectra of IR740-Cl methanolic solutions and Rdot-IR740-Cl (measurement time: 0.8 s).

Rdots were prepared based on previously published procedures (23, 24, 25). Here, IR780 (425,311, Sigma Aldrich) was used instead of IR783 (Fig. 1a) because IR780 is incorporated into beads more efficiently than IR783, while their Raman spectra are the same. A mixture of 150-µL, 2.5%, 40-nm PS beads (Invitrogen, C37232), 150-µL, 2%, F-127 (Invitrogen, P6867), and 300-µL Milli-Q were put in a 1.5-mL plastic tube. The mixture of 30-µL, 30-mM cyanine dye solution in DMSO and 270-µL tetrahydrofuran was added to the tube dropwise. The mixture was then vortexed for 10 min, followed by gentle agitation for another 10 min. Then, 14-mL Milli-Q water was added to the mixture to quench the dye loading. The mixture was then centrifuged (4000 rpm, 10 min) using 100 K MWCO filters (Millipore, UFC910024) to remove the solvents. The stained beads were washed with 15-mL Milli-Q water five times.

### Cell preparation

**Fig. 2.**
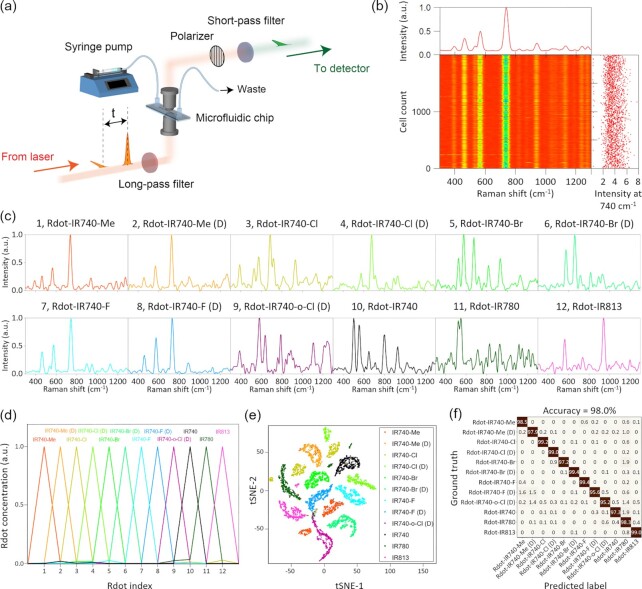
Multiplex flow cytometry with Raman tags. (a) Schematic of the FT-CARS flow cytometer. (b) Evidence for high reproducibility of the FT-CARS flow cytometry measurements. (c) Single-cell Raman spectra of MCF-7 cells stained by 12 different colors of Rdots. (d) Spectral unmixing of the single-cell spectra shown in Fig. 2b. (e) t-SNE projection of 11,777 single-cell Raman spectra obtained from MCF-7 cells stained by 12 different Rdots. (f) Confusion matrix for the classification of Rdots based on the obtained single-cell Raman spectra.

**Fig. 3.**
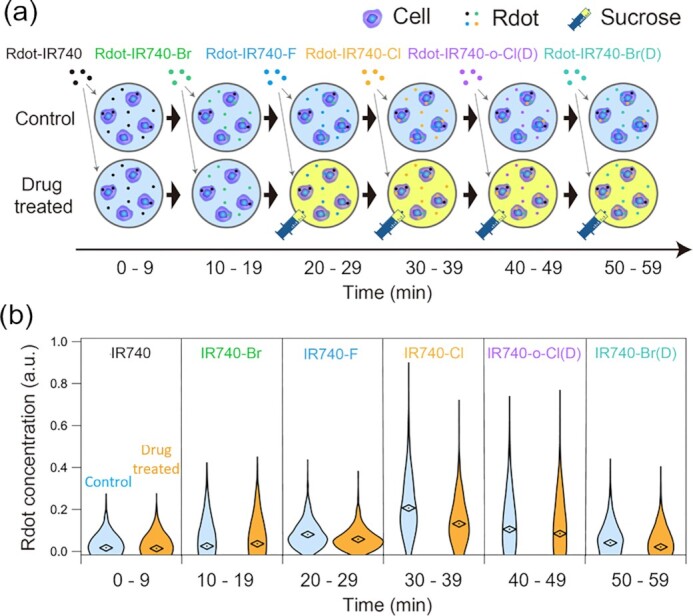
Large-scale time-course analysis of endocytosis by multiplex flow cytometry with Raman tags. (a) Experimental scheme of the cellular incorporation of Rdots. Cells were incubated with different Rdots to encode their time course by Rdots. For example, the number of Rdots incorporated in the period of 20 to 29 min was identifiable by measuring the Raman intensity of Rdot-IR740-CI from the cells. Unincorporated Rdots were removed between each time frame. After the incorporation, the cells were interrogated by our FT-CARS flow cytometer. (b) Concentrations of Rdots incorporated in each period within cells identified by the multiplex flow cytometer with Raman tags (*N* = 2,000 for each measurement). The concentrations of six Raman tags (the numbers of Rdots incorporated in the six periods) in a cell were determined from the single Raman spectrum obtained from the cell through the fitting analysis.

Breast cancer cells (MCF-7) were cultured using a D-MEM culture medium (FUJIFILM Wako, 044–29,765, +10% FBS, 1% P/S). Before the cell experiments, the cells were prepared to be half-confluent in 60-mm cell culture dishes. In the 12-color flow cytometry experiment (Fig. 2), cells were incubated at 37ºC for 2 h in a D-MEM culture medium containing Rdots at a concentration of 1 mg/mL. In the time-course endocytosis analysis experiment (Fig. 3), the cells of the control group were incubated at 37ºC for 10 min in a D-MEM culture medium containing Rdots at a concentration of 1 mg/mL for each color of Rdots. When switching the “color” of Rdots for the time-course analysis, unincorporated Rdots were removed by washing the cells with the culture medium three times. For the drug-treated group, sucrose was added to a culture medium at a concentration of 0.5 M in 20 to 60 min, while all other procedures are the same as the control group. For both experiments, unincorporated Rdots were removed by washing the cells with PBS five times after all the incubation procedures. After the removal, the cells were detached from the flask by treating them with an enzyme, trypsin/EDTA for 8 min at 37ºC. After the 8-min treatment, the cells were centrifuged at 1000 rpm for 3 min and the supernatant was removed. The cells were washed 3 times with PBS. After washing, the cells were used for the flow cytometry measurement.

### Estimation of the theoretical limit on the number of colors

**Fig. 4.**
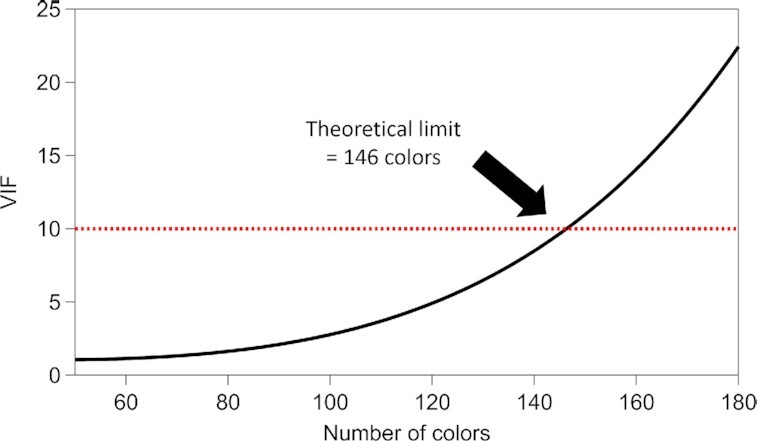
Theoretical estimation of the maximum number of colors. Our VIF analysis shows that it is feasible to achieve 140 colors with a single excitation laser and a single detector. VIF values were calculated from a data set composed of equally spaced single Gaussian peaks with 20 cm^−1^ FWHM (spectral resolution of the FT-CARS spectrometer) covering the entire spectral window (400 to 1,600 cm^−1^).

To estimate the theoretical limit of the number of colors that can be detected by our method, we calculated VIF values of artificially synthesized spectral sets, where each spectrum has a single Gaussian peak with a FWHM of 20 cm^−1^ (spectral resolution of our FT-CARS spectrometer). The peak positions were chosen such that they were equally spaced and covered the entire spectral region (400 to 1,600 cm^−1^). The maximum VIF value in each data set was plotted as a function of the numbers of spectra in each data set in Fig. 4.

### Estimation of the number of distinguishable Raman tags

We artificially synthesized spectral sets, where each spectrum has 10 Gaussian peaks with an FWHM of 20 cm^−1^ (spectral resolution of our FT-CARS spectrometer). The peaks were randomly placed in the detectable spectral region (400 to 1,600 cm^−1^) with random intensities. The spectrum with the highest VIF value was excluded from the spectral set. The VIF calculation and the spectral exclusion were repeated until the highest VIF value reached a value less than 10.

### Computational simulations on multiplex flow cytometry

To evaluate the performance of our method and compare it to that of fluorescence flow cytometry, we artificially synthesized phenotypic data for the classification of blood cells. In this simulation, we first prepared 29 cell types and determined the expression level of 20 CD markers for each cell type. The phenotypic expressions and CD markers used in this simulation are shown in Tables S1 and S2. Here, the averaged expression levels of the markers were determined based on mass cytometry results in ref. (15) (Fig. S3). The distributions of the expression levels were given by a lognormal distribution.

(i) For a Raman flow cytometry simulation, we artificially synthesized a spectral basis set consisting of 20 Raman tags with each spectrum having a single Gaussian peak with an FWHM of 20 cm^−1^ (spectral resolution of our FT-CARS spectrometer). The peak positions were chosen such that they were equally spaced and covered the entire spectral region (400 to 1,600 cm^−1^). We generated a 1,200-pixel Raman spectrum of the cell as a linear combination of the basis-set spectra, in which the weight coefficients were determined by the expression levels of the corresponding CD markers. We assumed that all the CD markers expressed on the cell surface were selectively stained by the corresponding Raman probes. After adding Gaussian noise with variable amplitudes to the spectra, we calculated the expression levels of the markers by the method presented in the “Multiplex flow cytometry with Raman tags” section.
(ii) For a fluorescence flow cytometry simulation with a single excitation laser, we artificially synthesized a spectral basis set of 20 fluorescent tags with each spectrum having a single Gaussian peak with an FWHM of 50 nm (typical spectral width of fluorescent tags). The peak positions were chosen such that they were equally spaced and covered the entire visible region (400 to 800 nm). Then, a 22-pixel fluorescence spectrum of the cell was generated based on the same procedure as (i) using the basis-set fluorescence spectra. After adding Gaussian noise with variable amplitudes to the spectra, we obtained the expression levels of the markers by a fitting calculation.
(iii) For a fluorescence flow cytometry simulation with five excitation lasers, we assumed that each dye has an excitation profile with a 50-nm-wide single Gaussian peak (typical spectral width of fluorescent tags). The excitation peak positions were chosen such that they were equally spaced and covered the entire spectral region longer than the excitation wavelength. The wavelengths of the five excitation lasers were assumed to perfectly match the five excitation peaks. Then, a spectrum of each cell was generated based on the same procedure as simulation (i), using fluorescence spectra as a basis set. The following part on the generation of cellular spectra and spectral unmixing are the same as simulation (ii).

After conducting the above procedures, we performed cell phenotyping with a support vector machine based on a cross-validation method. In this process, we used 80% of the data for training the model and classified the remaining 20% of the data with the trained model. This was repeated five times. The classification accuracy was calculated based on the above procedure and shown in Fig. S4 as a function of the noise amplitude normalized by the detection limit of each detection scheme. All the simulations above were performed with the software Igor pro 8.0.

## Results

### Raman tags for color-scalable flow cytometry

Fig. 1a shows the molecular structures and Raman spectra of 20 cyanine-based Raman tags, 7 and 13 of which were commercially available cyanine dyes and synthesized by us, respectively (see Supplementary Information for the complete synthesis procedures). We designed cyanine-based Raman tags based on cyanine dyes consisting of 7-methine structures, which show strong absorption around 740 nm. Since our FT-CARS spectrometer is pumped by a Ti: sapphire laser emitting 750 to 850-nm light (see Supplementary Information for the detailed schematic of the FT-CARS spectrometer), the Raman signals of the cyanine-based Raman tags were strongly enhanced by their electronic resonance between 740 and 820 nm. Our strategy to increase the number of colors without sacrificing the strong resonance Raman enhancement was to add substituents (−Me, −Cl, −Br, −F, −OMe) at the edge of the conjugated system of the IR740 skeleton (see Fig. 1a). Furthermore, using techniques from our previous reports (26, 27), hydrogens in the tetraene unit were substituted by deuteriums to shift Raman signals and double the number of the dyes without changing their absorption wavelengths. In fact, all the Raman tags show ∼10^5^ times stronger Raman signal compared to methanol (see Fig. S1). With the same strategy, we can further increase the number of colors by introducing different substituents (−I, −NO_2_, −CF_3_) and different isotopes (^13^C, ^15^N) at different positions.

To use the synthesized cyanine-based Raman tags for multiplex measurements, their Raman spectra need to be linearly independent. To quantitatively evaluate the linear independence of the Raman spectra of our 20 Raman tags, we calculated their variance inflation factor (VIF) values (Fig. 1b). VIF is a measure of multicollinearity in a set of multidimension data. It is known that a data set with a high VIF value is expressed by a linear combination of the other variables. Here, when the VIF is applied to Raman spectroscopy, with the VIF value of the optical spectrum of a molecule exceeding a certain threshold, its spectrum can be reproduced by a linear superposition of the spectra of other molecules (28). Although the threshold value is dependent on the signal quality in the case of spectroscopy, we set the value at 10 as it is commonly used in statistics (28). Among the VIF values of our Raman tags, IR740-OMe shows the highest value of 10.07, while the other Raman tags show VIF values lower than 10. These results show the linear independence of the spectra of 19 cyanine-based Raman dyes.

Next, we evaluated the limit of detection (LoD) for FT-CARS spectroscopy with each cyanine-based Raman tag by measuring the FT-CARS spectra of IR740-Cl at various concentrations (Fig. 1c, circles). For a spectral acquisition time of 420 µs, which is the typical duration of a single-cell measurement in FT-CARS flow cytometry, the LoD of IR740-Cl was found to be 8 µM. Although this value is much lower than the typical LoD in coherent Raman spectroscopy in the nonresonance regime, further enhancement was needed for meaningful biological applications such as surface antigen marker detection. For this purpose, we enclosed the Raman tags in 40-nm polystyrene (PS) nanoparticles to prepare 12 different Rdots based on the swelling-diffusion technique (25) (see Supplementary Information for the detailed procedure of preparing the Rdots). As shown in Fig. 1c, the LoD of the Rdots was found to be 12 nM, which is >600 times lower than the LoD of the original cyanine-based Raman tag. The Raman spectra obtained from free IR740-Cl molecules dissolved in methanol and Rdots composed of IR740-Cl are shown in Fig. 1d. While the relative peak intensities of the dyes and the Rdots are slightly different, their overall spectral profiles are preserved upon the incorporation of the tags into PS nanoparticles. This implies that the prepared Rdots retained low spectral multicollinearity seen in the free Raman tags dissolved in methanol. The absence of detectable background signals from PS can be explained by the strong electronic resonance enhancement (enhancement factor > 10^5^) of the Raman signal from the dye molecules. Considering that the concentration of the styrene group in the Rdots is higher than those of the dye molecules in the Rdots by a factor of ∼100, the detectable Raman signal of PS is weaker than the Raman signals of Rdots by a factor of >10^3^.

### Multiplex flow cytometry with Raman tags

To demonstrate multiplex flow cytometry with Raman tags, we performed measurements of MCF-7 breast cancer cells stained by 12 different Rdots with our FT-CARS flow cytometer (Fig. 2a). The Rdots were incorporated into the cells through endocytosis by culturing them in media containing the prepared Rdots (see Supplementary Information for the detailed procedure of the cell preparation). For each color, Raman spectra of 1,000 single cells were obtained at an event rate of 20 to 50 cells/s with a spectral acquisition time of ∼750 µs/cell (18 spectra/cell) and an incident optical power of 150 mW. The Raman spectra of the cells stained by IR740-Me (*N* = 2,000), their intensities at 740 cm^−1^, and the average spectrum are shown in Fig. 2b, which shows the high reproducibility of our FT-CARS flow cytometry measurements. Typical Raman spectra obtained from the single cells stained by the 12 different Rdots are shown in Fig. 2c, in which the spectral profiles are shown to be distinct from each other. To quantify the concentrations of Rdots in the cells, the obtained single-cell spectra were fitted with a model spectrum expressed by $\ \mathop \sum \limits_i {C}_i{S}_i$, where ${C}_i$ and ${S}_i$, the relative concentration and reference Raman spectrum of the *i*-th Rdot, respectively. Fig. 2d shows the ${C}_i$ values obtained from the spectra of 12 single cells stained by 12 different Rdots, showing that each single-cell spectrum was well reproduced solely by one reference spectrum. To demonstrate the applicability of the present method to large-scale analysis, all the obtained spectra (*N *= 11,777) were projected onto a two-dimensional (2D) space using the t-SNE algorithm (Fig. 2e), which is an algorithm to project the data from a high-dimensional space to a low-dimensional space by minimizing Kullback–Leibler divergence between the data distributions in both spaces (29) (see the raw data available in Supplementary data). In the t-SNE plot, the cells stained with the different Rdots are well separated, indicating that the spectral resolvability was retained at a single-cell level. More quantitatively, the obtained 11,777 spectra were classified into one color of Rdot based on the maximum ${C}_i$ values. A total of 11,544 out of 11,777 spectra were found to be assigned to the correct Rdots (accuracy = 98.0%) (Fig. 2f).

### Large-scale time-course analysis of cellular endocytosis

To show the capability of our method to study cellular activity on a large scale, we applied it to a large-scale time-course analysis of cellular endocytosis via multiplex flow cytometry with Raman tags. Specifically, MCF-7 breast cancer cells were consecutively incubated in culture media containing six different Rdots (Fig. 3a). By using different Rdots in different periods of the incubation, the activity of endocytosis at different times was encoded in each cell’s Rdot concentration, which was decoded by Raman spectral measurements. Between 20 and 60 min after the beginning of the incubation, an endocytosis inhibitor, sucrose, was added to the culture media to suppress the endocytosis activity (Fig. 3a). After the incubation, unincorporated Rdots were removed, and the cells were detached from the flask using trypsin/EDTA for multiplex flow cytometry measurements. In each condition (control, sucrose), 2,000 cells were measured at event rates of 40 to 50 cells/s with the incident optical power of 150 mW. The concentrations of Rdots in the cells were determined by fitting a superposition of the basis spectra to the obtained single-cell Raman spectra. In the control group, where no inhibitor was added, the endocytosis activity was maximized from 20 to 39 min and then decayed after 40 min (Fig. 3b). This trend is consistent with the previously reported endocytosis dynamics (30). For the groups treated with sucrose (0.5 M) from 20 to 59 min, suppressed endocytosis activity was observed immediately after the drug treatment (20 to 29 min). The suppression continued until the 59th min, which is also consistent with a previous report on the efficacy of sucrose inhibition (30, 31). Our results show that the power of multiplex flow cytometry with Raman tags combined with the spectral encoding of cellular dynamics enabled large-scale analysis of endocytosis. In this proof-of-principle demonstration, we used six colors for observing endocytosis in six different time periods, but our method is extendable with a much larger number of colors, allowing for measurements with longer time periods.

## Discussion

The method presented here can be further improved in the following directions. First, the number of colors can be increased by synthesizing new cyanine dyes in the present scheme. While we added substituents to the edge of the IR740 skeleton for multiplexing in this study, >100 different tags can be synthesized by using different positions (>30 positions) and skeletons (>5 skeletons). Unlike the nitrile- and alkyne-based Raman tags synthesized for the silent region, a randomly synthesized cyanine-based Raman tag probably has a low VIF value due to the broadband nature of our FT-CARS flow cytometer. This easy-to-design property is central to the color scalability in our Raman-tag-based multiplex flow cytometry approach. In fact, the theoretical limit of our method on the number of colors was calculated to be 146 with a single excitation laser based on a simple simulation where each spectrum has a single Gaussian peak (Fig. 4). With a more realistic simulation using multipeak spectra, it is expected that 80 distinguishable Raman tags are obtained by synthesizing 100 different molecules (Fig. S2). Moreover, our simulation of classifying 29 different phenotypic expressions using Raman and fluorescent tags (Figs. S3 and S4) shows that a classification accuracy comparable to fluorescence flow cytometry equipped with five excitation lasers can be achieved with our method only with a single excitation laser. Second, the detection sensitivity of Rdots can be enhanced by optimizing the staining procedure to import more dyes into one particle. However, increasing the number of dyes per bead does not always increase its Raman signal intensity in the present method. This is because the intermolecular interactions between the Raman tag molecules significantly change their electronic resonance. By appropriately designing spacer molecules as demonstrated in the development of ultrabright fluorescence nanoparticles (32), it would be possible to enclose more Raman tags in a unit volume. Third, the present method can be used for phenotyping cells by integrating with immunostaining. As Rdots can easily be functionalized by NHS-ester groups followed by bond formation to antibodies (23, 24), Rdot-based immunostaining would readily be performed. Phenotyping of cells with the number of colors comparable to mass cytometry is feasible in a nondestructive manner with this approach.

## Supplementary Material

pgad001_Supplemental_File

## Data Availability

All data are included in this manuscript and its supplementary information files.
