# Supplementary material for: Color-scalable flow cytometry with Raman tags
Source: PNAS Nexus. 2023 Jan 14;2(2):pgad001. doi: 10.1093/pnasnexus/pgad001 (PMC9950787; doi:10.1093/pnasnexus/pgad001)
Supplement: pgad001_Supplemental_File [file pgad001_supplemental_file.docx]

**Supplementary Information for**

Color-scalable flow cytometry with Raman tags

Ryo Nishiyama, Kotaro Hiramatsu*, Shintaro Kawamura, Kosuke Dodo, Kei Furuya, Julia Gala de Pablo, Shigekazu Takizawa, Wei Min, Mikiko Sodeoka, and Keisuke Goda*

Kotaro Hiramatsu

Email: [hiramatsu@chem.s.u-tokyo.ac.jp](mailto:hiramatsu@chem.s.u-tokyo.ac.jp)

Keisuke Goda

Email: [goda@chem.s.u-tokyo.ac.jp](mailto:goda@chem.s.u-tokyo.ac.jp)

**This PDF file includes:**

Supplementary text

Figures S1 to S22

Tables S1 to S2

Schemes S1 to S10

SI References

**
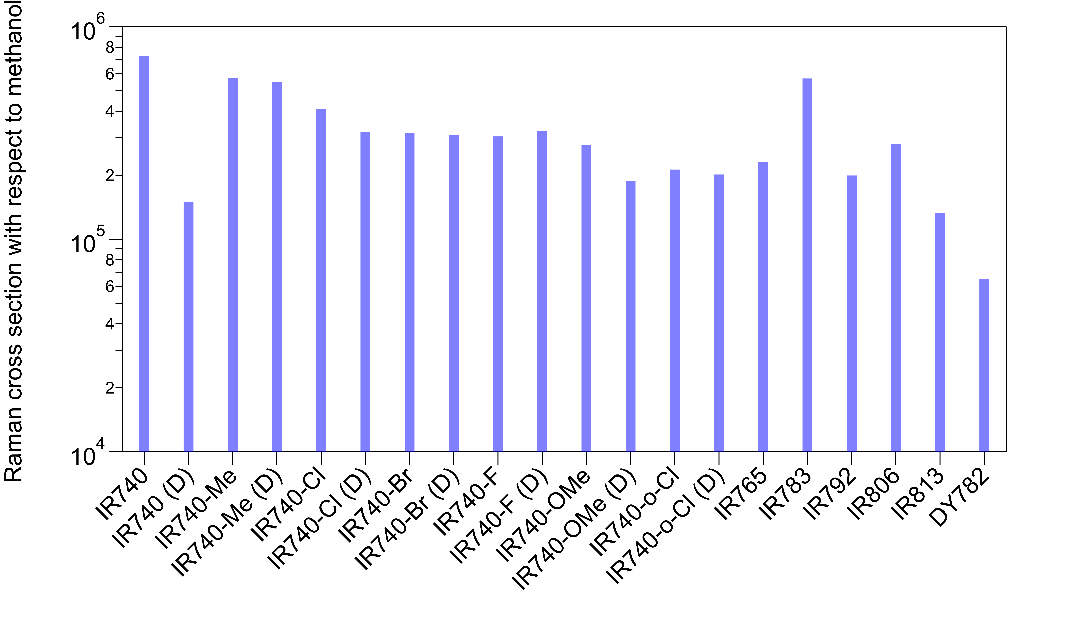
**

**Fig. S1. Relative Raman cross section of the Raman tags**. Relative Raman cross sections of the Raman tags with respect to that of the 1037-cm^-1^ peak of methanol are shown. The values were evaluated using our FT-CARS spectrometer.


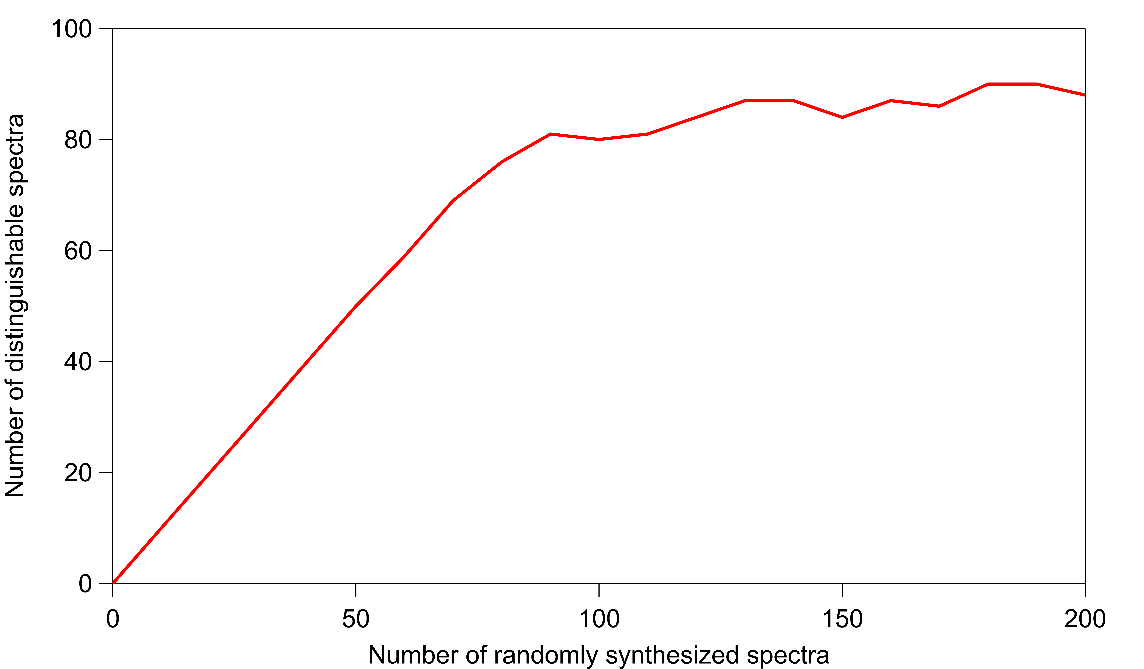


**Fig. S2. Estimation of the number of distinguishable Raman tags among randomly synthesized dye molecules.** We synthesized 10 - 200 Raman spectra consisting of 10 Gaussian peaks with random intensities and peak positions. The VIF values of those spectra were calculated. The spectrum with the highest VIF value was excluded from the spectral set until the highest VIF value reached a value less than 10. The number of distinguishable Raman tags is defined as the number of spectra remaining after all the above procedures.


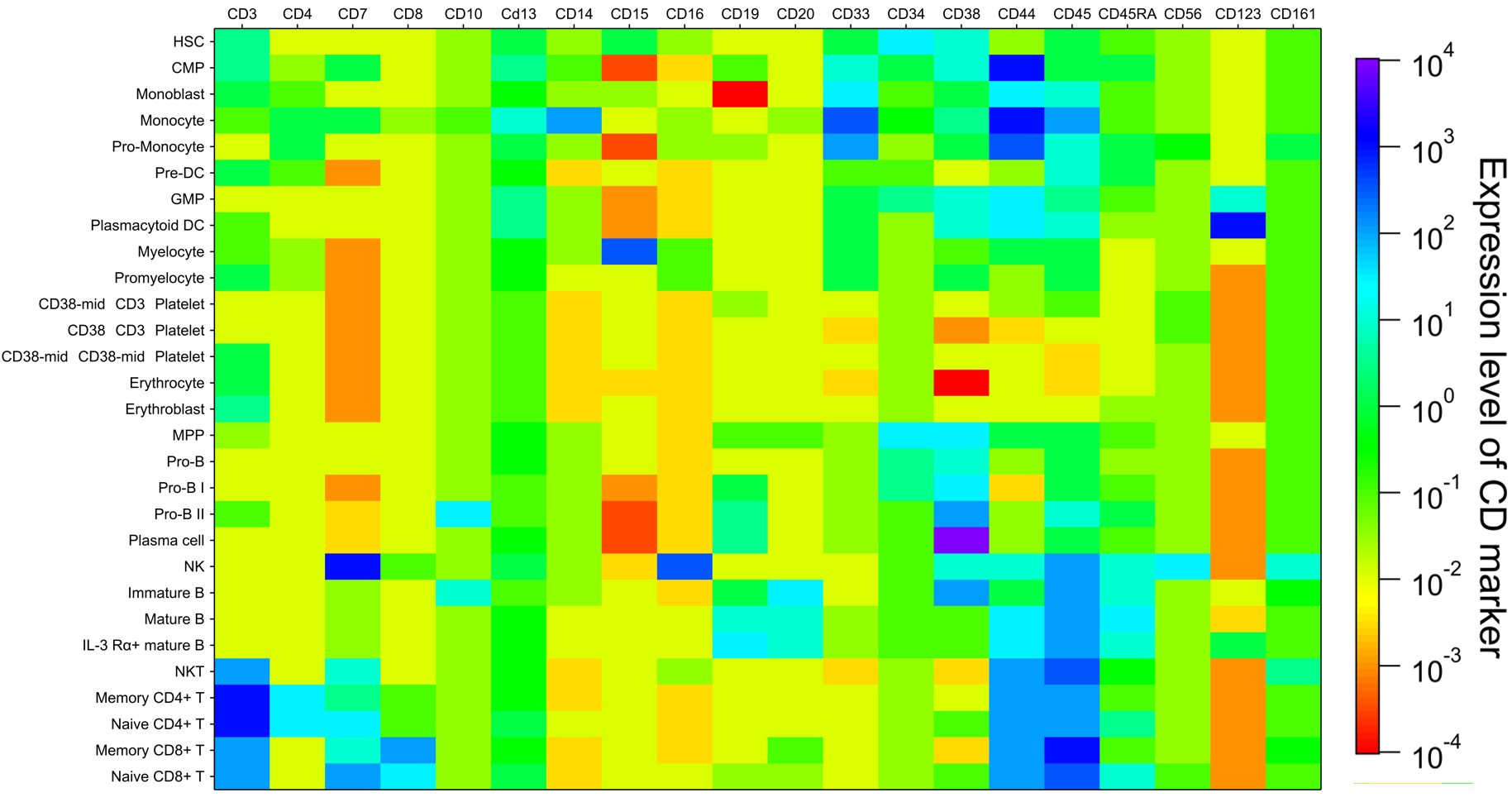


**Fig. S3. Phenotypic expression levels of blood cells using CD markers.** Mean expression levels of the CD markers on blood cells are shown here. The data were used for the computational simulations on multiplex flow cytometry.


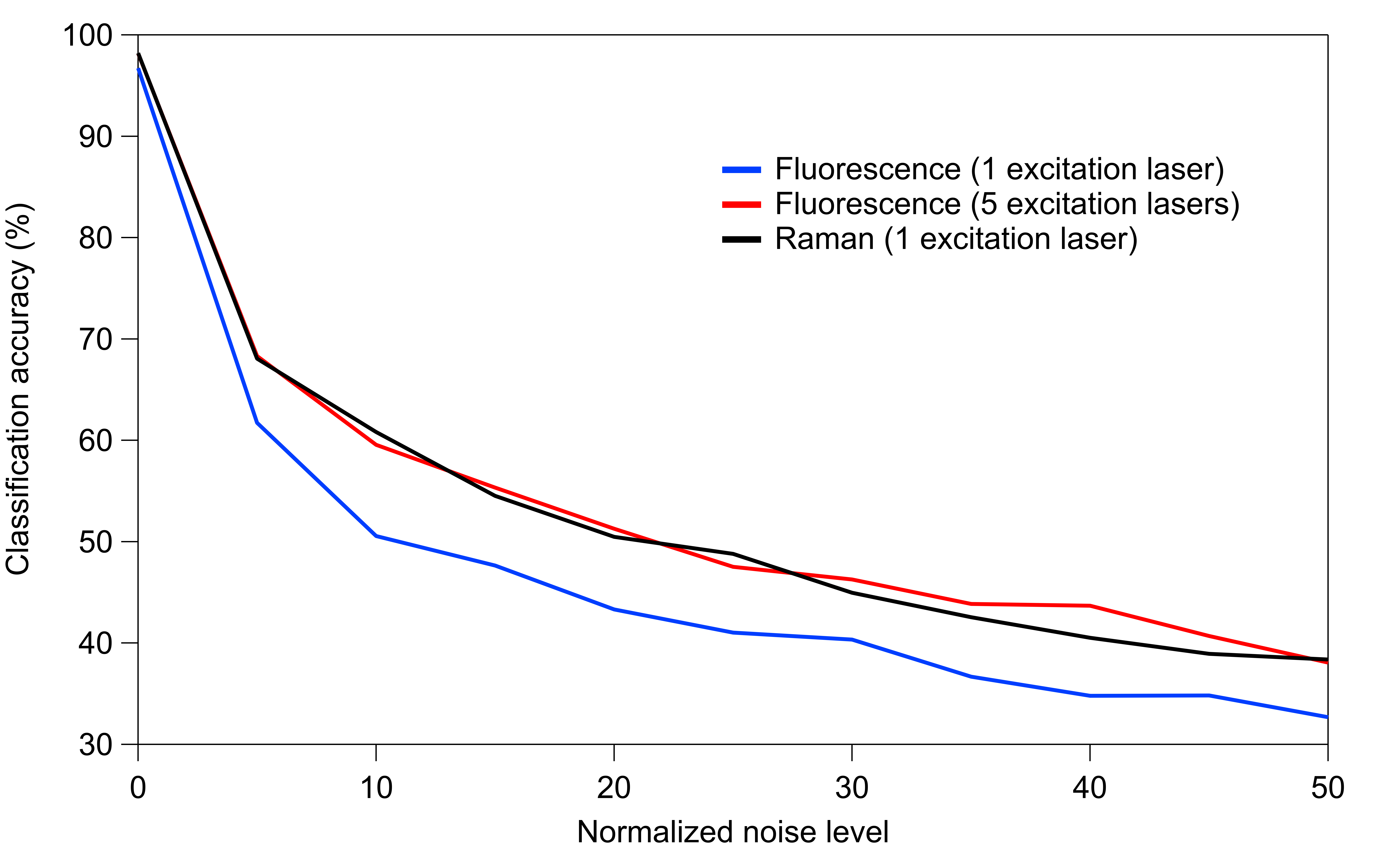


**Fig. S4. Computational simulations on multiplex flow cytometry.** The result of the computational simulations of 20-color flow cytometry are shown here. The phenotyping accuracy for 29 different immune cell types are plotted as a function of the noise level normalized by the detection limit in each detection scheme. The blue, red, and black lines represent the simulation result for fluorescence flow cytometry with a single excitation laser, fluorescence flow cytometry with five excitation lasers, and FT-CARS flow cytometry with a single excitation laser, respectively.


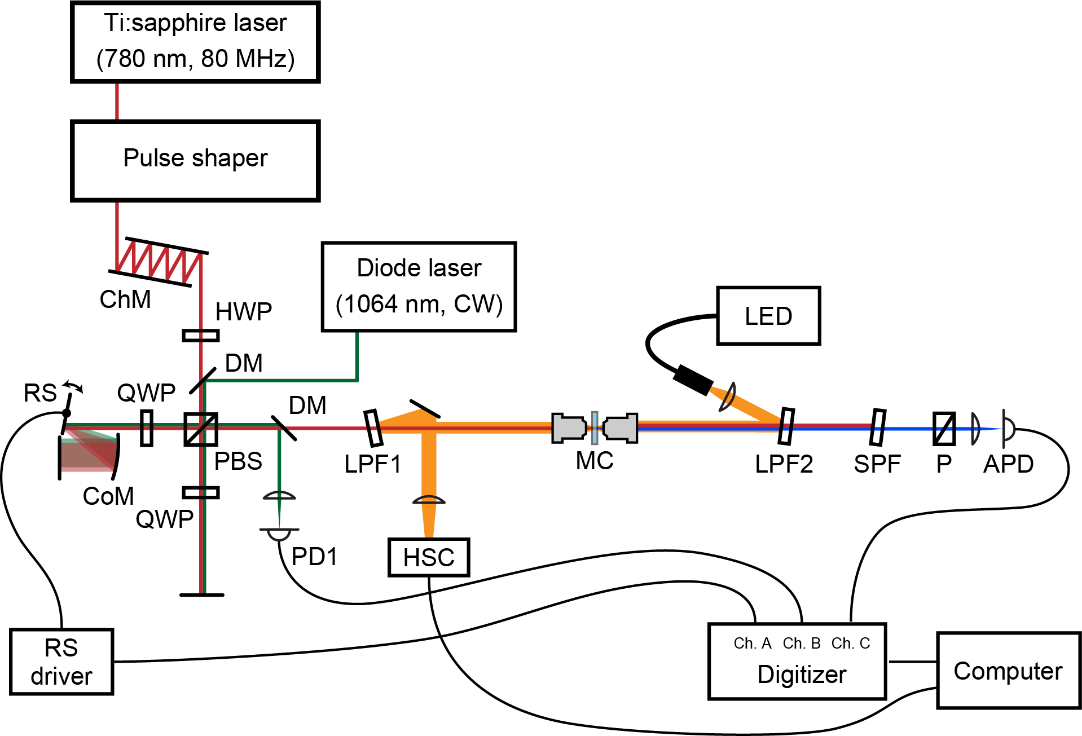


**Fig. S5. Schematic of our FT-CARS flow cytometer**. CW: continuous wave; ChM: Chirped mirror pair; HWP: Half-wave plate; DM: dichroic mirror; PBS: Polarizing beam splitter; QWP: Quarter-wave plate; RS: Resonant scanner; CoM: Concave mirror; PD1: InGaAs photodiode; HSC: High-speed camera; LPF1: Long-pass filter with a cutoff wavelength of 750 nm; MC: microfluidic chip, LPF2: Long-pass filter with a cutoff wavelength of 650 nm; SPF: Short-pass filter with a cutoff wavelength of 750 nm; P: Polarizer; APD: Avalanche photodiode; SM: Spatial mask; PD2: Si photodiode.


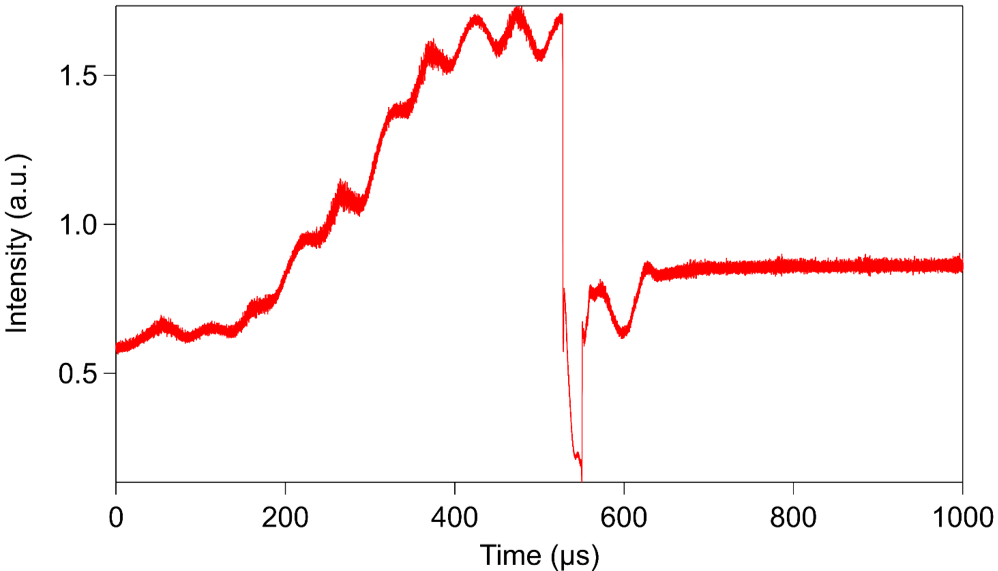


**Fig. S6. Typical time-domain FT-CARS signal removed by gating.**

**
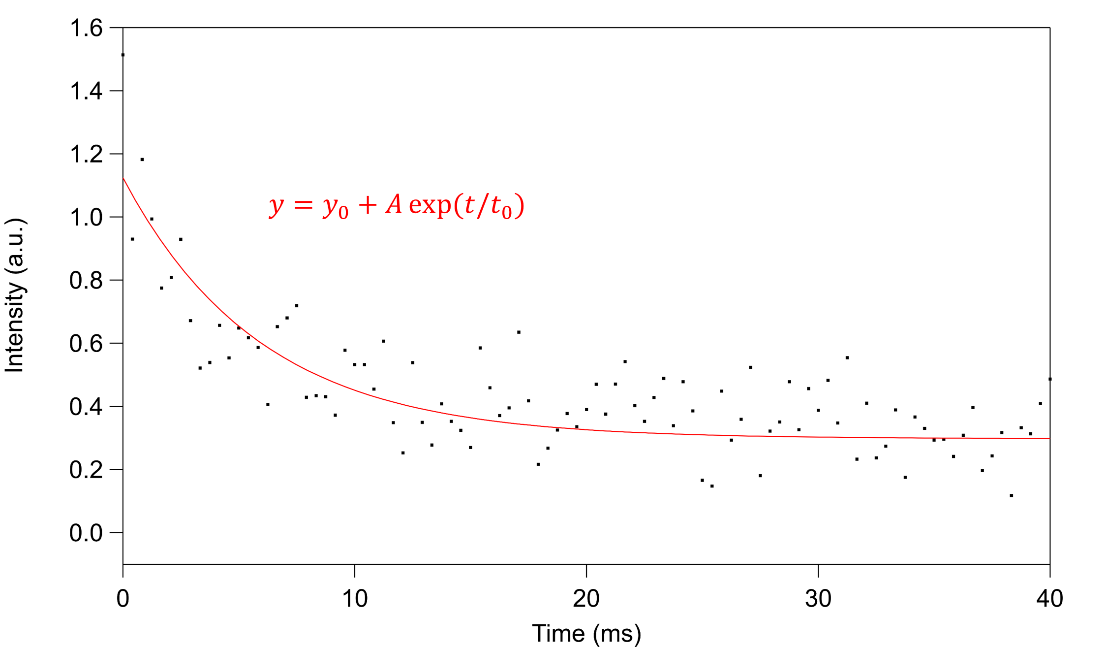
**

**Fig. S7. Photostability of the Raman tag.** Data from our photobleaching measurement of the Raman tag (IR740) under 150-mW irradiation is shown. The red curve was obtained by fitting analysis with an exponential function: $y_{0}=2.98, A=0.82, t_{0}=5.94 (ms)$.

**Table. S1. Phenotypic expressions used in our computational simulations on multiplex flow cytometry.** The phenotypic expressions of the cells used for immunophenotyping in the computational simulations are shown here.

| HSC | CD38-mid-CD3-Platelet | NK |
| --- | --- | --- |
| CMP | CD38-CD3-Platelet | Immature B |
| Monoblast | CD38-mid-CD3-mid-Platelet | Mature B |
| Monocyte | Erythrocyte | IL-3 Rα+ mature B |
| Pro-Monocyte | Erythroblast | NKT |
| Pre-DC | MPP | Memory CD4+ T |
| GMP | Pro-B | Naïve CD4+ T |
| Plasmacytoid DC | Pre-B I | Memory CD8+ T |
| Myelocyte | Pre-B II | Naïve CD8+ T |
| Promyelocyte | Plasma cell |  |

**Table. S2. CD markers used in our computational simulations on multiplex flow cytometry.** The CD markers used for immunophenotyping in the computational simulations are shown here.

| CD3 | CD4 | CD7 | CD8 | CD10 | CD13 | CD14 | CD15 | CD16 | CD19 |
| --- | --- | --- | --- | --- | --- | --- | --- | --- | --- |
| CD20 | CD33 | CD34 | CD38 | CD44 | CD45 | CD45RA | CD56 | CD123 | CD161 |

**Supplementary Information Text**

**Synthetic procedure of Raman tags**

**Synthesis of 5-substituted 2,3,3- trimethylindolenine species 2-6**

Compounds **2-6** were synthesized according to the literature procedure (33). 4-Substituted phenylhydrazine hydrochloride derivative (*p*-Tolylhydrazine Hydrochloride, Sigma Aldrich, T40606 / 4-Chlorophenylhydrazine Hydrochloride, Sigma Aldrich, C65807 / 4-Bromophenylhydrazine Hydrochloride, FUJIFILM Wako, 326-26891 / 4-Fluorophenylhydrazine Hydrochloride, FUJIFILM Wako, 358-21751 / 4-Methoxyphenylhydrazine Hydrochloride, Sigma Aldrich, 152137, 3.0 g) was dissolved in glacial acetic acid (25 mL) in a round-bottom flask and the mixture was stirred at 80 ºC for 30 min. Then, 3-methyl-2-butanone (FUJIFILM Wako, 131-03783, 3 mol. equiv.) was added. The mixture was refluxed for 15-20 h and cooled to room temperature. The resulting mixture was concentrated with a rotary evaporator to remove acetic acid. The concentrated oil was diluted with 25-mL dichloromethane (25 mL), washed with water three times, and dried over MgSO_4_. The solvent was then removed with a rotary evaporator to obtain the indolenine product. All of the compounds (**2–6**) were used for the next step without purification.


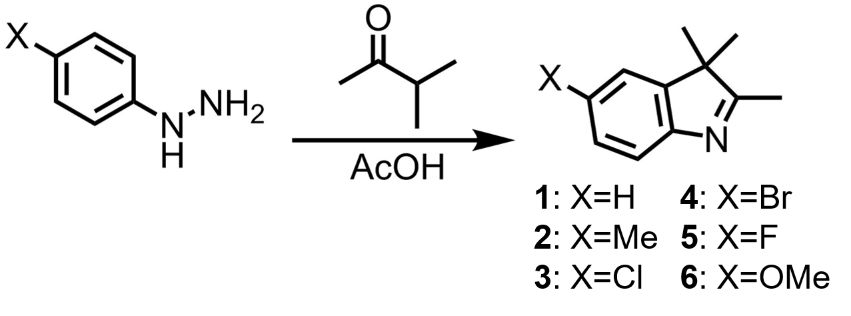


**Scheme S1.** Synthesis of 5-substituted indolenine species **1**-**6**.

**Synthesis of 5-substituted 1,2,3,3-tetramethylindolium salts 8-12**

Compounds **8-12** were synthesized according to the literature procedure (33). To a solution of 5-substituted 2,3,3-trimethylindolenine (**2-6**) in anhydrous acetonitrile (10 mL), iodomethane (FUJIFILM Wako, 142-04191, 3.0 eq.) was added. The mixture was refluxed (100 ºC) for 12–15 h. After cooling to room temperature, the acetonitrile was removed with a rotary evaporator. The crude product was dissolved in methanol, and then cold diethyl ether was added to precipitate the product. The solid was filtered out and dried to obtain compounds **8**-**12**. Compound **7** was purchased from Tokyo Chemical Industry Co., Ltd. (T0766).


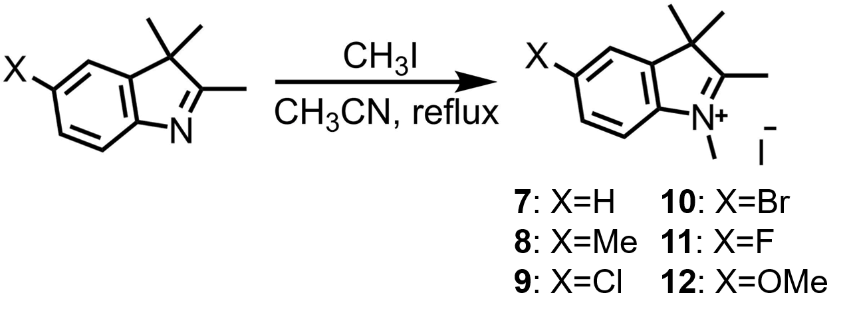


**Scheme S2.** Synthesis of 5-substituted indolenium salts **7-12**.

**Synthesis of 7-chloro 2,3,3- trimethylindolenine 13**

Compound **13** was synthesized according to the literature procedure (33). 2-Chlorophenylhydrazine hydrochloride (3.0 g) was added to glacial acetic acid (25mL) in a round-bottom flask and the mixture was stirred at 80 ºC for 30 min. Then, 3-methyl-2-butanone (3.0 eq., 5.34 mL) was added to the solution. The mixture was refluxed for 4 h and cooled to room temperature. The resulting mixture was concentrated with a rotary evaporator to remove acetic acid. The resulted oil was diluted with dichloromethane (25 mL), washed with water three times, and dried over MgSO_4_. The solvent was then removed with a rotary evaporator to obtain 0.93 g of compound **13,** which was used for the next step without purification.


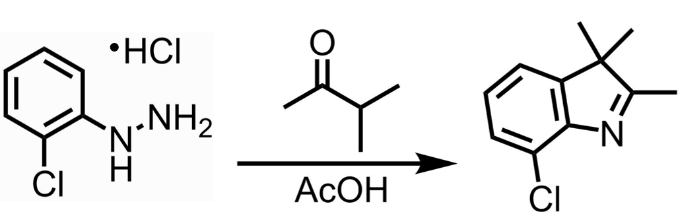


**Scheme S3.** Synthesis of 7-chloro indolenine **13**.

**Synthesis of 7-chloro 1,2,3,3-tetramethylindolium salt 14**

Compound **14** was synthesized according to the literature procedure (33). To a solution of compound **13** (0.93g) in anhydrous acetonitrile (10 mL), iodomethane (3.0 eq., 1.0 mL) was added. The mixture was refluxed (100 ºC) for 12 h. After cooling to room temperature, acetonitrile was removed with a rotary evaporator. The crude product was dissolved in methanol, and then cold diethyl ether was added to precipitate the product. The solid was filtered out and dried to obtain 0.35 g of compound **14** (Yield: 22 %).


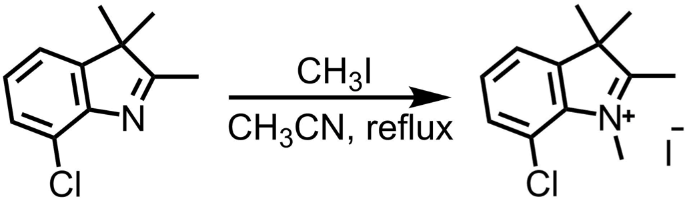


**Scheme S4.** Synthesis of 7-chloro indolenium salt **14**.

**Synthesis of 5-substituted symmetric cyanine dyes 16-20**

Compounds **15**–**20** were synthesized according to the literature procedure (34). In a test tube, 100 mg of the indolenium salt (**8-12**) and glutaconaldehydedianil hydrochloride (FUJIFILM Wako, 053324, 0.5 eq.) were allowed to settle. Then, acetic anhydride (0.25 mL) and triethylamine (3.0 eq.) were added. The mixture was warmed to 60 ºC and stirred for 2 h. After cooling to room temperature, dichloromethane and water (1:1 ratio) were added to the reaction mixture. Extraction with dichloromethane was repeated three times. After washing the solution with water, dichloromethane was removed with a rotary evaporator. Flash column chromatography of the crude product on silica gel (5–10% methanol in dichloromethane) afforded the compounds **16-20**. Compound **15** was purchased from Sigma Aldrich (252034).


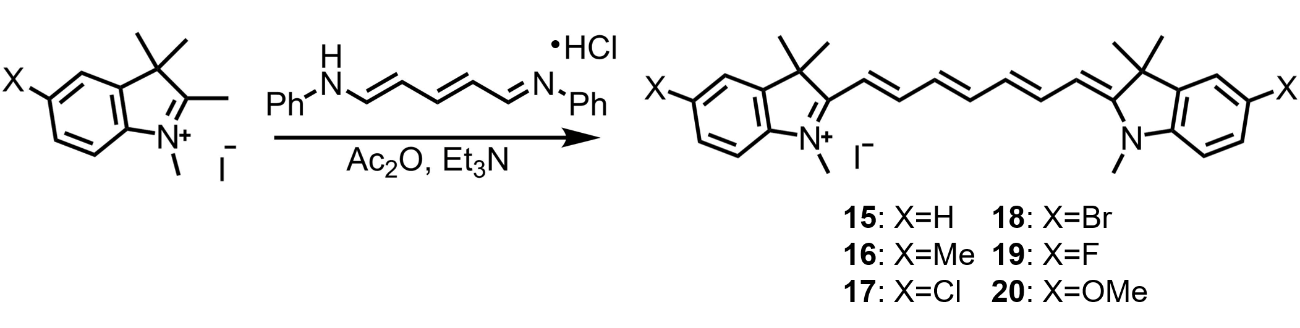


**Scheme S5.** Synthesis of symmetric cyanine dyes **15**-**20**.

**Synthesis of 7-chloro symmetric cyanine dye 21**

Compound **21** was synthesized according to the literature procedure (34). In a test tube, 100 mg of the indolenium salt **14** and 42 mg of glutaconaldehydedianil hydrochloride (0.5 eq.) were allowed to settle. Then, acetic anhydride (0.25 mL) and triethylamine (3.0 eq., 62.3 µL) were added. The mixture was warmed to 60 ºC and stirred for 2 h. After cooling to room temperature, dichloromethane and water (1:1 ratio) were added to the reaction mixture. Extraction with dichloromethane was repeated three times. After washing the solution with water, dichloromethane was removed with a rotary evaporator. Flash column chromatography of the crude product on silica gel (5–10% methanol in dichloromethane) afforded 58 mg (Yield: 62%) of pure product **21**.


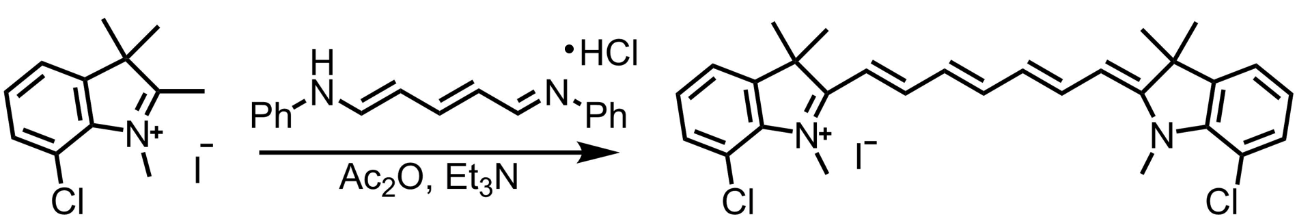


**Scheme S6.** Synthesis of 7-chloro symmetric cyanine dye **21**.

**Synthesis of deuterated Zincke salt 22**

Compound **22** was synthesized according to the literature procedure (35). 1-Chloro-2,4-dinitrobenzene (2.0 g, Tokyo Chemical Industry, C0162) was dissolved in acetone (10 mL) with stirring in a flask. Pyridine-d5, 99.5% (810 µL, FUJIFILM Wako, 163-28381) was added to the solution, and the mixture was refluxed (75 ºC) for 19 h and cooled to room temperature. The reaction mixture was then filtered to obtain a white solid. The solid was washed with pentane and 2.2 g of compound **22** was obtained (Yield: 38%).


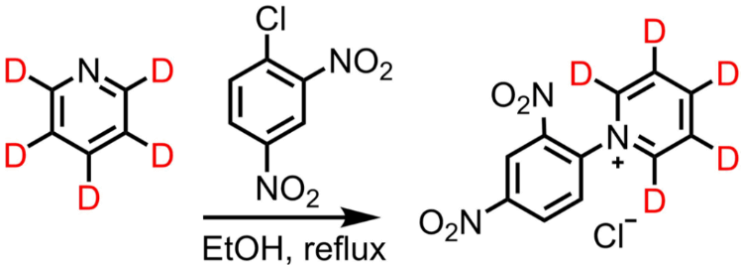


**Scheme S7.** Synthesis of deuterated Zincke salt **22**.

**Synthesis of 1,2,3,4,5-deuterated glutaconaldehydedianil hydrochloride 23**

The compound **23** was also synthesized based according to the literature procedure (36). Compound **22** (2.0 g) was dissolved in 80% aqueous ethanol (18 mL). A solution of aniline (1.4 mL) in 80% aqueous ethanol (18 ml) was slowly added to the mixture. After stirring for 70 min, the reaction mixture was filtered to obtain a solid. The solid was dissolved in methanol and diethyl ether was added to precipitate the product. The solid was washed with diethyl ether and dried to afford 0.58 g of compound **23** (Yield: 29%).


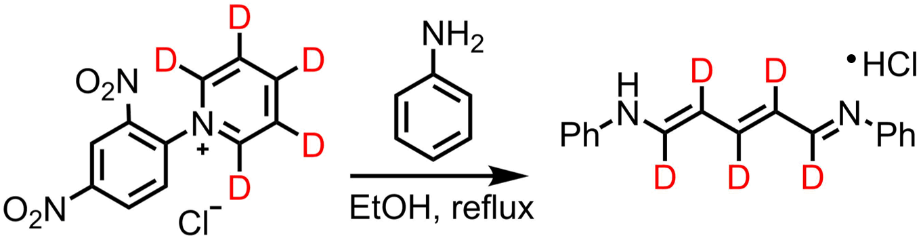


**Scheme S8.** Synthesis of deuterated glutaconaldehydedianil hydrochloride **23**.

**Synthesis of with deuterated 5-substituted symmetric cyanine dyes 24-29**

Compounds **24**–**29** were synthesized according to the literature procedure (34). In a test tube, 100 mg of the indolenium salt (**8-12**) and compound **23** were allowed to settle. Then, acetic anhydride (0.25 mL) and triethylamine (3.0 eq.) were added. The mixture was warmed to 60 ºC and stirred for 2 h. After cooling to room temperature, dichloromethane and water (1:1 ratio) were added to the reaction mixture. Extraction with dichloromethane was repeated three times. After washing the solution with water, dichloromethane was removed with a rotary evaporator. Flash column chromatography of the crude product on silica gel (5–10% methanol in dichloromethane) afforded the pure target products **24**-**29**.


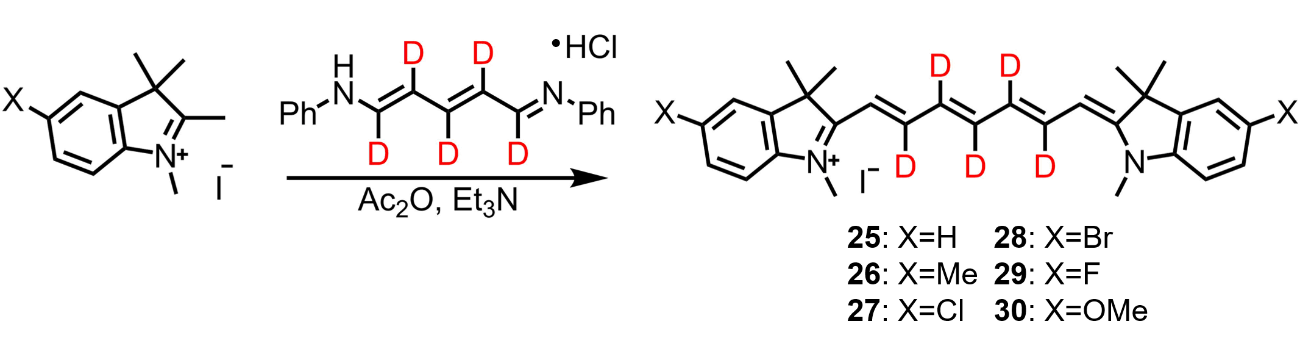


**Scheme S9.** Synthesis of deuterated 5-substituted symmetric cyanine dyes **25**–**29**

**Synthesis of deuterated 7-chloro symmetric cyanine dye 30**

Compound **30** was synthesized according to the literature procedure (34). In a test tube, 100 mg of the indolenium salt **14** and compound **23** were allowed to settle. Then, acetic anhydride (0.25 mL) and triethylamine (3 eq., 62.5 µL) were added. The mixture was warmed to 60 ºC and stirred for 2 h. After cooling to room temperature, dichloromethane and water (1:1 ratio) were added to the reaction mixture. Extraction with dichloromethane was repeated three times. After washing the solution with water, dichloromethane was removed with a rotary evaporator. Flash column chromatography of the crude product on silica gel (5–10% methanol in dichloromethane) afforded 60 mg (Yield: 66%) of pure product **30**.


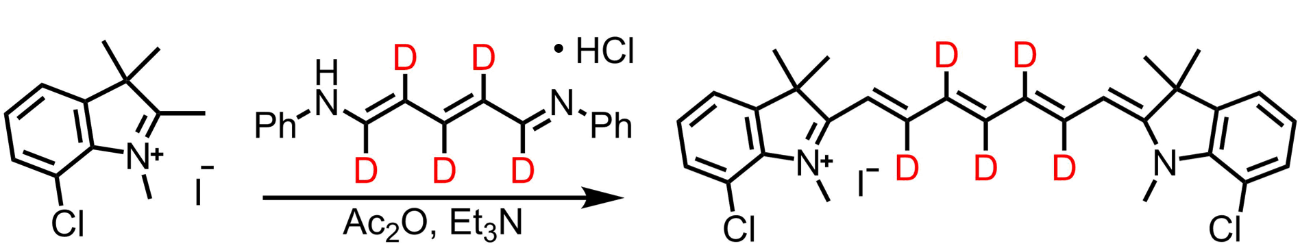


**Scheme S10.** Synthesis of deuterated 7-chloro symmetric cyanine dye **30**.

**Characterization of Raman tags**

2-(7-(1,3,3,5-tetramethylindolin-2-ylidene)-1,3,5-heptatrienyl)-1,3,3,5-tetramethylindolium iodide (IR740-Me, **16**)

Yield 89%; ^1^H-NMR (600 MHz, DMSO-D6) δ 7.81 (t, *J* = 13.4 Hz, 2H), 7.69 (m, 1H), 7.38 (s, 2H), 7.24 (d, *J* = 7.8 Hz, 2H), 7.19 (d, *J* = 7.8 Hz, 2H), 6.48 (m, 2H), 6.25 (d, *J* = 13.4 Hz, 2H), 3.54 (s, 6H), 2.35 (s, 6H), 1.60 (s, 12H); MS (ESI^+^), m/z (relative intensity, %): 439.2 (M^+^, 7), 438.2 (52), 437.2 (100).

2-(7-(5-chloro-1,3,3-trimethylindolin-2-ylidene)-1,3,5-heptatrienyl)-5-chloro-1,3,3-trimethylindolium iodide (IR740-Cl, **17**)

Yield 82%; ^1^H-NMR (600 MHz, DMSO-D6) δ 7.85 (m, 2H), 7.77-7.72 (m, 3H), 7.44 (d, *J* = 8.5 Hz, 2H), 7.36 (d, *J* = 8.5 Hz, 2H), 6.53 (m, 2H), 6.30 (d, *J* = 13.4 Hz, 2H), 3.55 (s, 6H), 1.62 (s, 12H); MS (ESI^+^), m/z (relative intensity, %): 482.1 (M^+^, 4), 481.1 (13), 480.1 (19), 479.1 (68), 478.1 (34), 477.1 (100).

2-(7-(5-bromo-1,3,3-trimethylindolin-2-ylidene)-1,3,5-heptatrienyl)-5-bromo-1,3,3-trimethylindolium iodide (IR740-Br, **18**)

Yield 35%; ^1^H-NMR (401 MHz, DMSO-D6) δ 7.89-7.82 (m, 4H), 7.75 (m, 1H), 7.57 (dd, *J* = 8.5, 2.1 Hz, 2H), 7.31 (d, *J* = 8.5 Hz, 2H), 6.53 (m, 2H), 6.30 (d, *J* = 13.8 Hz, 2H), 3.54 (s, 6H), 1.62 (s, 12H); MS (ESI^+^), m/z (relative intensity, %): 571.0 (M^+^, 2), 570.0 (16), 569.0 (55), 568.0 (33), 567.0 (100), 566.0 (17), 565.0 (54)

2-(7-(5-fluoro-1,3,3-trimethylindolin-2-ylidene)-1,3,5-heptatrienyl)-5-fluoro-1,3,3-trimethylindolium iodide (IR740-F, **19**)

Yield 33%; ^1^H-NMR (401 MHz, DMSO-D6) δ 7.83 (m, 2H), 7.72 (m, 1H), 7.56 (dd, *J* = 8.3, 2.8 Hz, 2H), 7.37 (dd, *J* = 8.7, 4.1 Hz, 2H), 7.23 (td, *J* = 8.7, 2.8 Hz, 2H), 6.50 (m, 2H), 6.27 (d, *J* = 13.7 Hz, 2H), 3.55 (s, 6H), 1.62 (s, 12H); MS (ESI^+^), m/z (relative intensity, %): 447.2 (M^+^, 8), 446.2 (49), 445.2 (100).

2-(7-(5-methoxy-1,3,3-trimethylindolin-2-ylidene)-1,3,5-heptatrienyl)-5-methoxy-1,3,3-trimethylindolium iodide (IR740-OMe, **20**)

Yield 32%; ^1^H-NMR (401 MHz, DMSO-D6) δ 7.77 (m, 2H), 7.65 (m, 1H), 7.27 (d, *J* = 8.7 Hz, 2H), 7.23 (d, *J* = 2.3 Hz, 2H), 6.94 (dd, *J* = 8.7, 2.3 Hz, 2H), 6.43 (m, 2H), 6.20 (d, *J* = 13.7 Hz, 2H), 3.78 (s, 6H), 3.53 (s, 6H), 1.61 (s, 12H); MS (ESI^+^), m/z (relative intensity, %): 471.2 (M^+^, 7), 470.2 (38), 469.2 (100).

2-(7-(7-chloro-1,3,3-trimethylindolin-2-ylidene)-1,3,5-heptatrienyl)-7-chloro-1,3,3-trimethylindolium iodide (IR740-o-Cl, **21**)

Yield 62%; ^1^H-NMR (400 MHz, DMSO-D6) δ 7.91 (m, 2H), 7.80 (m, 1H), 7.55 (d, J = 7.3 Hz, 2H), 7.39 (d, J = 7.8 Hz, 2H), 7.20 (dd, J = 7.8, 7.3 Hz, 2H), 6.59 (m, 2H), 6.40 (d, J = 13.7 Hz, 2H), 3.88 (s, 6H), 1.62 (s, 12H); ^13^C NMR (101 MHz, DMSO-d6): δ 27.37, 34.66, 48.36, 104.88, 115.97, 121.62, 125.70, 126.60, 130.87, 138.25, 144.42, 151.73, 156.61, 172.76; MS (ESI^+^), m/z (relative intensity, %): 482.1 (M^+^, 3), 481.1 (13), 480.1 (21), 479.1 (68), 478.1 (32), 477.1 (100).

*N*-(1,2,3,4,5-pentadeuterio-5-(phenylimino)penta-1,3-dienyl)aniline (**23**)

Yield 11%; ^1^H-NMR (401 MHz, DMSO-D6) δ 12.07 (s, 2H), 7.41 (m, 8H), 7.19 (m, 2H); MS (ESI^+^), m/z (relative intensity, %): 250.1 (M^+^, 3), 251.1 (30), 252.1 (6), 253.1 (46), 254.1 (100), 255.1 (18), 256.1 (2).

2-(2,3,4,5,6-pentadeuterio-7-(1,3,3-trimethylindolin-2-ylidene)-1,3,5-heptatrienyl)-1,3,3-trimethylindolium iodide (IR740 (D), **24**)

Yield 64%, ^1^H-NMR (401 MHz, DMSO-D6) δ 7.53 (d, *J* = 7.4 Hz, 2H), 7.37-7.31 (m, 4H), 7.17 (td, *J* = 7.4, 1.2 Hz, 2H), 6.27 (s, 2H), 3.54 (s, 6H), 1.58 (s, 12H); MS (ESI^+^), m/z (relative intensity, %): 416.3 (M^+^, 5), 415.3 (31), 414.3 (100), 413.3 (4).

2-(2,3,4,5,6-pentadeuterio-7-(1,3,3,5-tetramethylindolin-2-ylidene)-1,3,5-heptatrienyl)-1,3,3,5-tetramethylindolium iodide (IR740-Me (D), **25**)

Yield 68%, ^1^H-NMR (401 MHz, DMSO-D6) δ 7.38 (s, 2H), 7.25-7.18 (m, 4H), 6.24 (s, 2H), 3.54 (s, 6H), 2.34 (s, 6H), 1.59 (s, 12H); MS (ESI^+^), m/z (relative intensity, %): 444.2 (M^+^, 10), 443.2 (57), 442.2 (100), 441.2 (9).

2-(2,3,4,5,6-pentadeuterio-7-(5-chloro-1,3,3-trimethylindolin-2-ylidene)-1,3,5-heptatrienyl)-5-chloro-1,3,3-trimethylindolium iodide (IR740-Cl (D), **26**)

Yield 71%, ^1^H-NMR (401 MHz, DMSO-D6) δ 7.72 (d, *J* = 2.1 Hz, 2H), 7.44 (dd, *J* = 8.4, 2.1 Hz, 2H), 7.36 (d, *J* = 8.4 Hz, 2H), 6.29 (s, 2H), 3.54 (s, 6H), 1.62 (s, 12H); MS (ESI^+^), m/z (relative intensity, %): 487.1 (M^+^, 4), 486.1 (14), 485.1 (23), 484.1 (75), 483.1 (39), 482.1 (100), 481.1 (5), 480.1 (2), 479.1 (5), 478.1 (2), 477.1 (7).

2-(2,3,4,5,6-pentadeuterio-7-(5-bromo-1,3,3-trimethylindolin-2-ylidene)-1,3,5-heptatrienyl)-5-bromo-1,3,3-trimethylindolium iodide (IR740-Br (D), **27**)

Yield 68%, ^1^H-NMR (401 MHz, DMSO-D6) δ 7.83 (d, *J* = 1.8 Hz, 2H), 7.57 (dd, *J* = 8.7, 1.8 Hz, 2H), 7.31 (d, *J* = 8.7 Hz, 2H), 6.29 (s, 2H), 3.54 (s, 6H), 1.62 (s, 12H); MS (ESI^+^), m/z (relative intensity, %): 576.0 (M^+^, 3), 575.0 (16), 574.0 (52), 573.0 (42), 572.0 (100), 571.0 (35), 570.0 (51), 569.0 (9).

2-(2,3,4,5,6-pentadeuterio-7-(5-fluoro-1,3,3-trimethylindolin-2-ylidene)-1,3,5-heptatrienyl)-5-fluoro-1,3,3-trimethylindolium iodide (IR740-F (D), **28**)

Yield 40%, ^1^H-NMR (401 MHz, DMSO-D6) δ 7.55 (dd, *J* = 8.3, 2.6 Hz, 2H), 7.36 (dd, *J* = 8.7, 4.1 Hz, 2H), 7.23 (td, *J* = 8.7, 2.8 Hz, 2H), 6.26 (s, 2H), 3.55 (s, 6H), 1.62 (s, 12H); MS (ESI^+^), m/z (relative intensity, %): 452.2 (M^+^, 7), 451.2 (47), 450.2 (100), 449.2 (2).

2-(2,3,4,5,6-pentadeuterio-7-(5-methoxy-1,3,3-trimethylindolin-2-ylidene)-1,3,5-heptatrienyl)-5-methoxy-1,3,3-trimethylindolium iodide (IR740-OMe(D), **29**)

Yield 67%, ^1^H-NMR (401 MHz, DMSO-D6) δ 7.26 (d, *J* = 8.7 Hz, 2H), 7.23 (d, *J* = 2.5 Hz, 2H), 6.94 (dd, *J* = 8.7, 2.5 Hz, 2H), 6.19 (s, 2H), 3.78 (s, 6H), 3.53 (s, 6H), 1.61 (s, 12H); MS (ESI^+^), m/z (relative intensity, %): 476.2 (M+, 7), 475.2 (37), 474.2 (100), 473.2 (3).

2-(2,3,4,5,6-pentadeuterio-7-(7-chloro-1,3,3-trimethylindolin-2-ylidene)-1,3,5-heptatrienyl)-7-chloro-1,3,3-trimethylindolium iodide (IR740_o-Cl (D), **30**)

Yield 66%, ^1^H-NMR (400 MHz, DMSO-D6) δ 7.55 (dd, *J* = 7.8, 0.9 Hz, 2H), 7.39 (dd, *J* = 7.8, 0.9 Hz, 2H), 7.20 (t, *J* = 7.8 Hz, 2H), 6.39 (s, 2H), 3.87 (s, 6H), 1.62 (s, 12H); MS (ESI^+^), m/z (relative intensity, %): 487.1 (M^+^, 3), 486.1 (13), 485.1 (22), 484.1 (68), 484.1 (68), 483.1 (35), 482.1 (100), 481.1 (3).

**
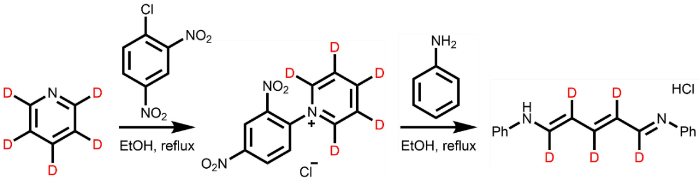

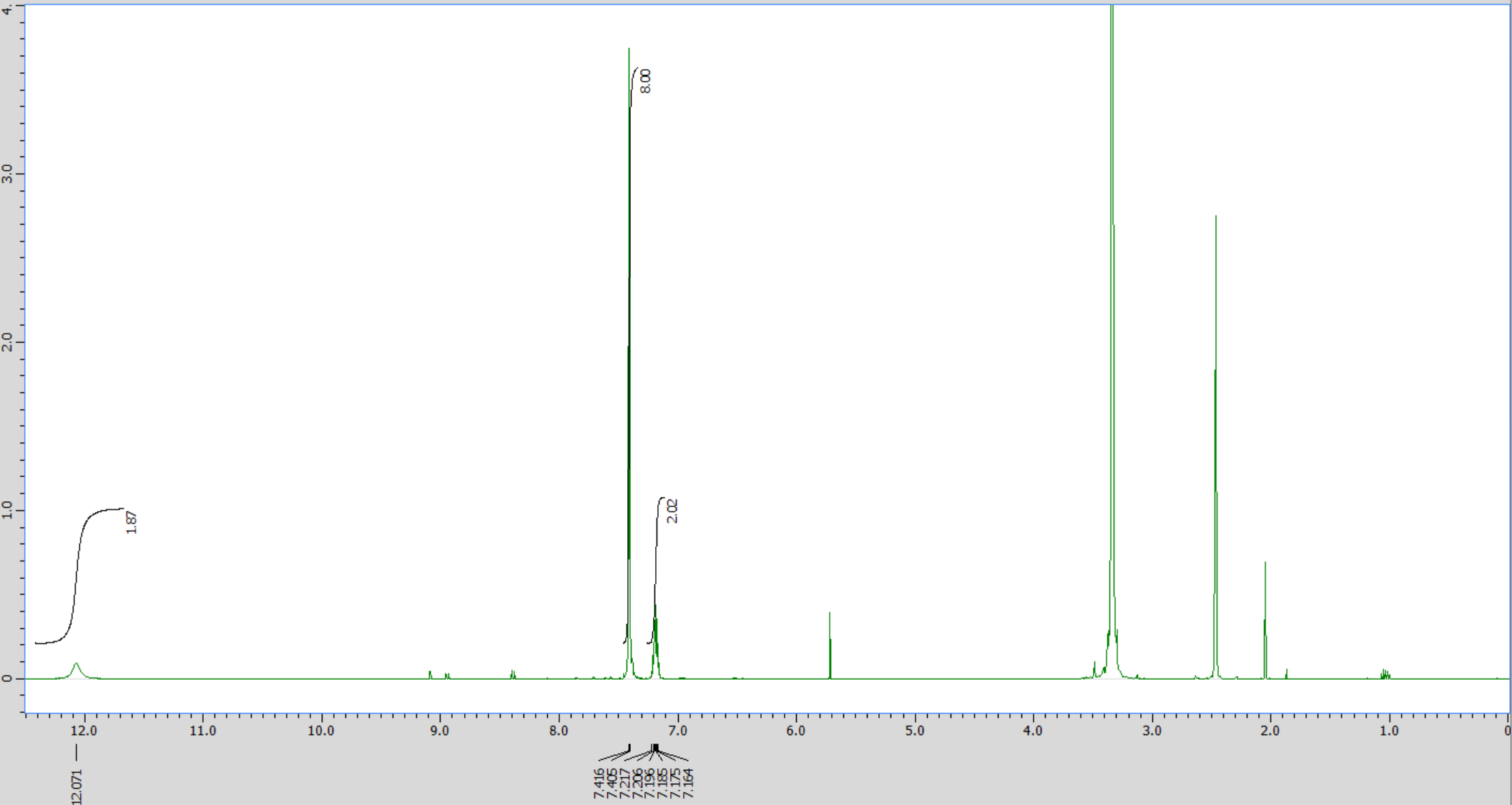
**

**Fig. S8. ^1^H NMR spectrum of Glutaconaldehydedianil Hydrochloride (D) (DMSO-d6)**

**
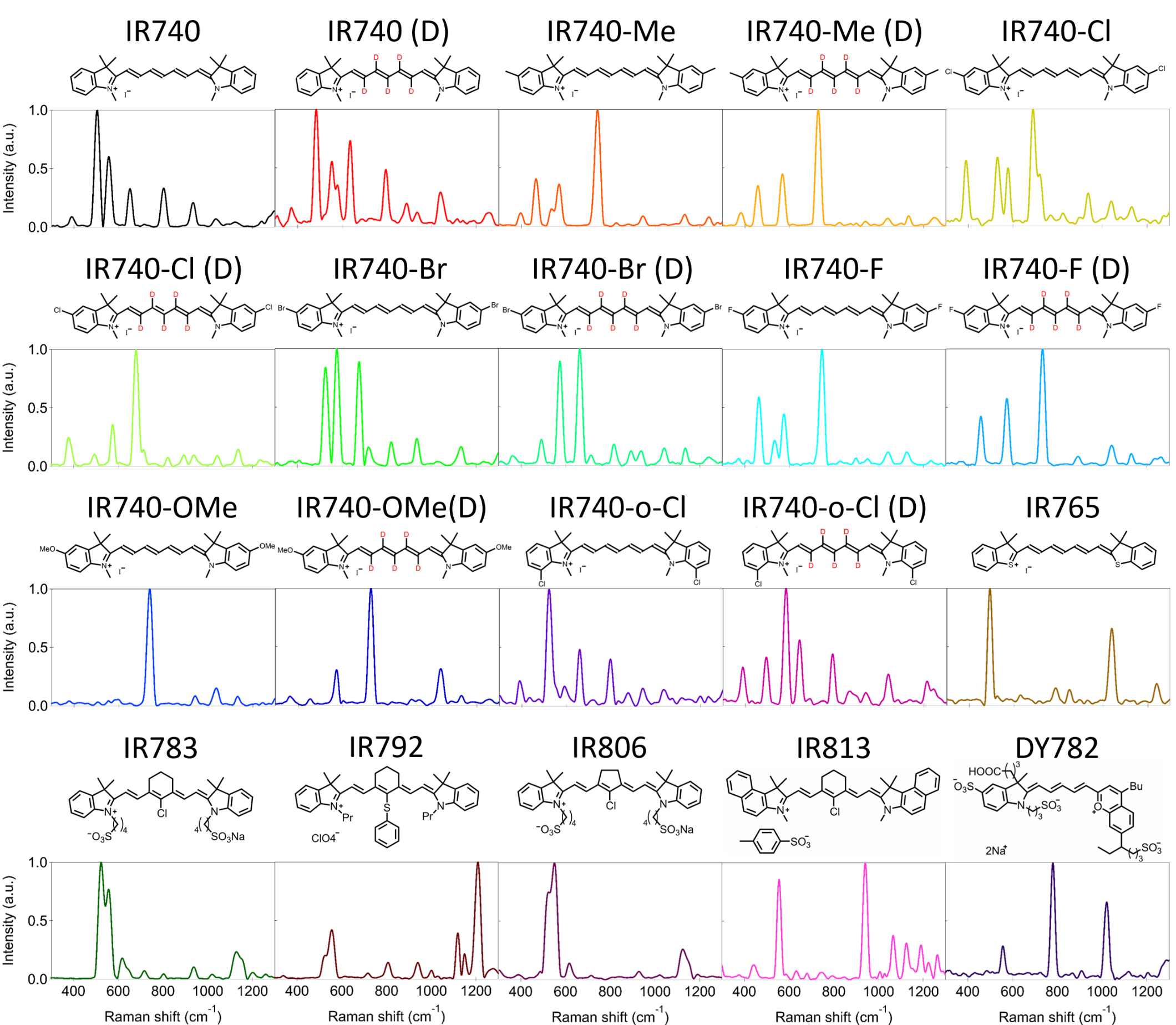
**

**
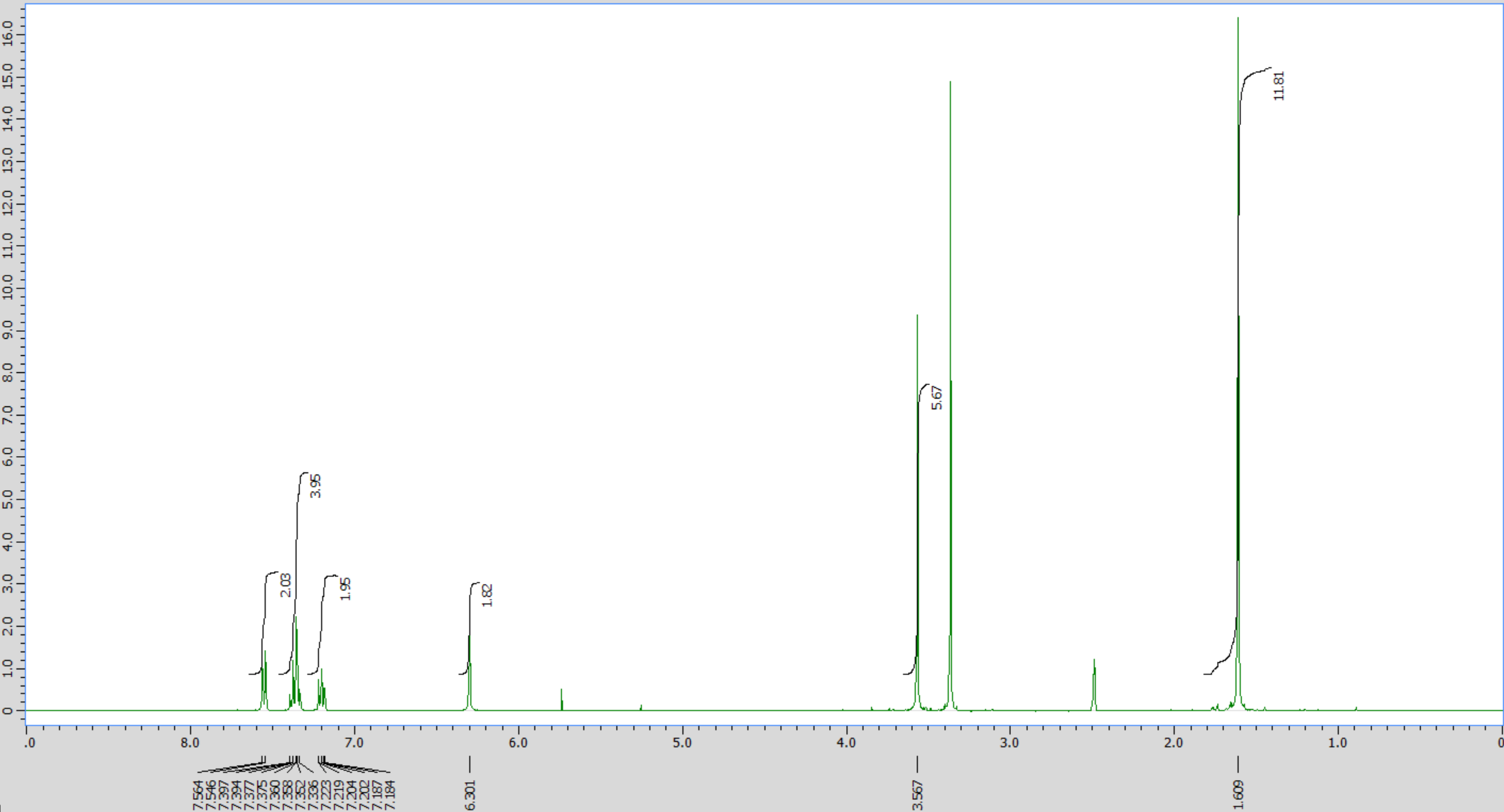
**

**Fig. S9. ^1^H NMR spectrum of IR740-H (D) (DMSO-d6)**

**
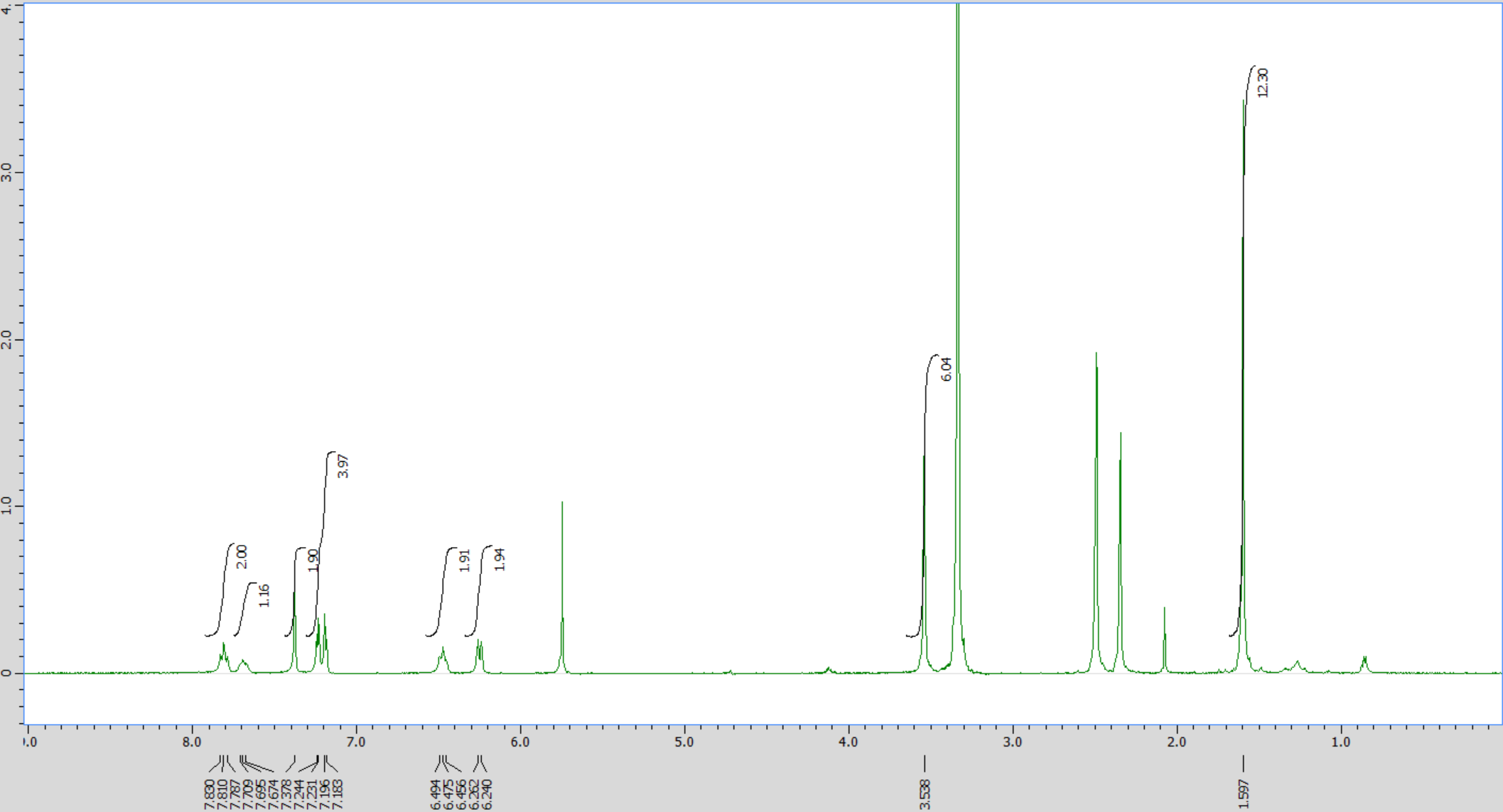

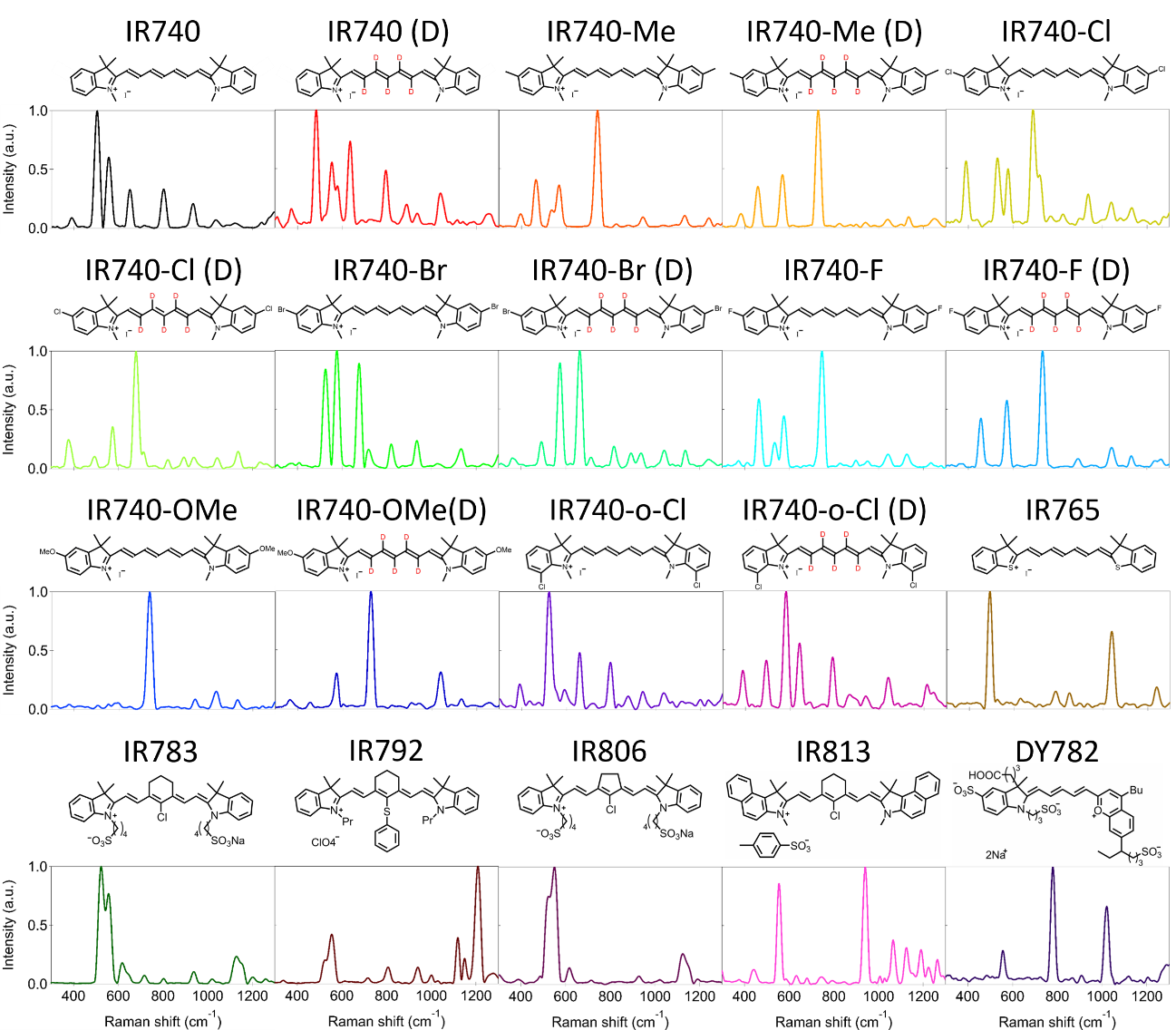
**

**Fig. S10. ^1^H NMR spectrum of IR740-Me (DMSO-d6)**

**
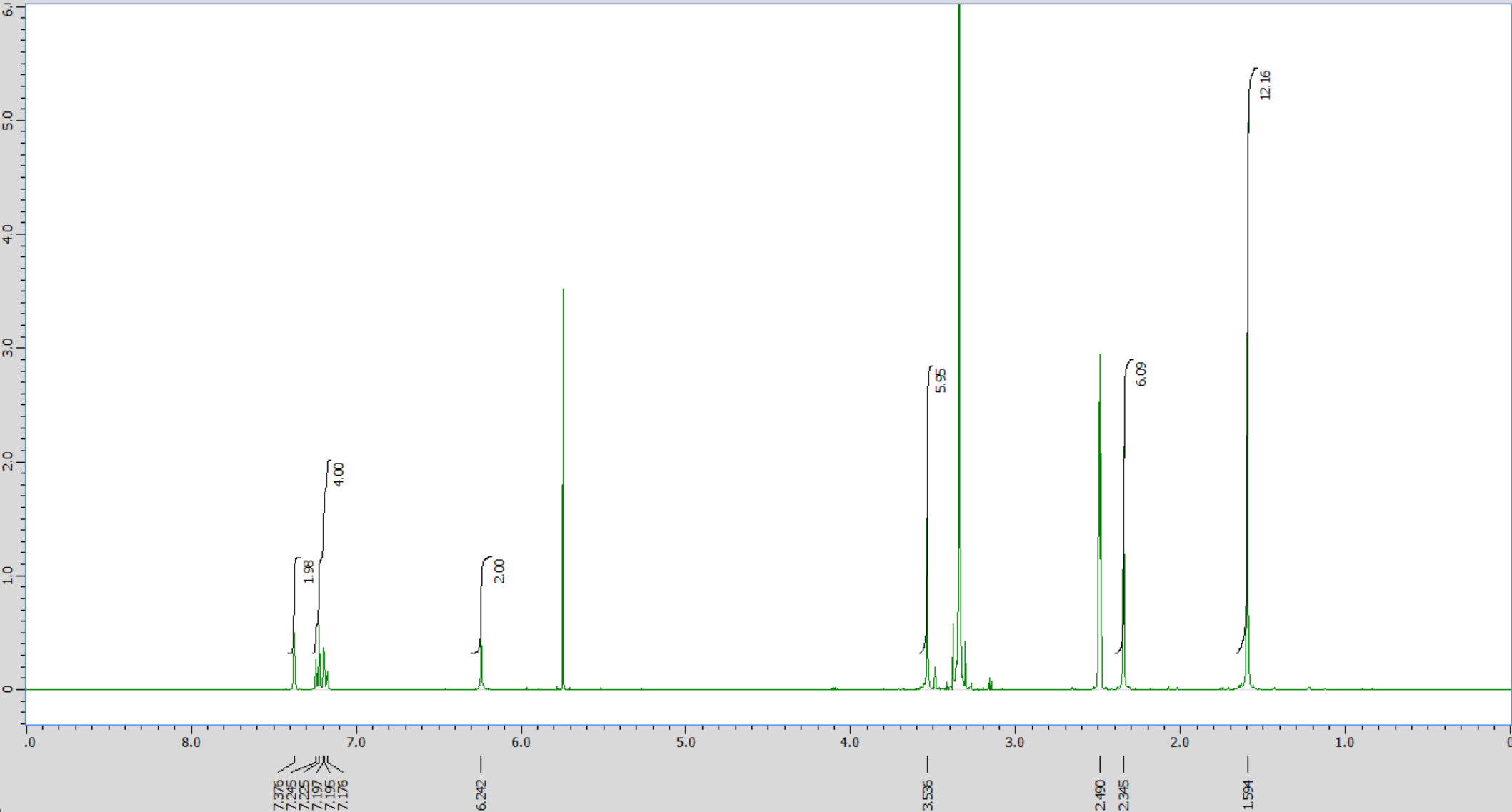

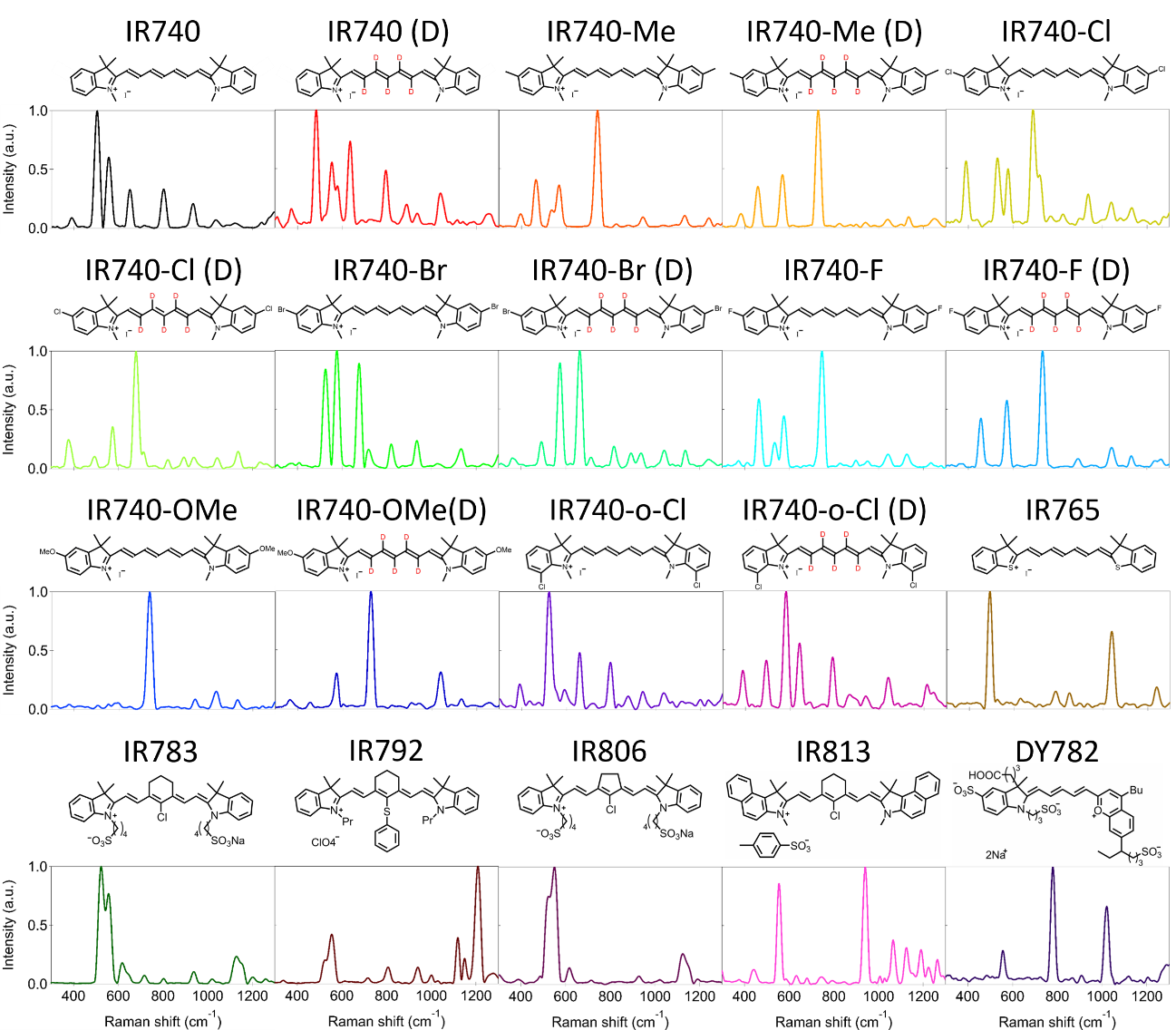
**

**Fig. S11. ^1^H NMR spectrum of IR740-Me (D) (DMSO-d6)**

**
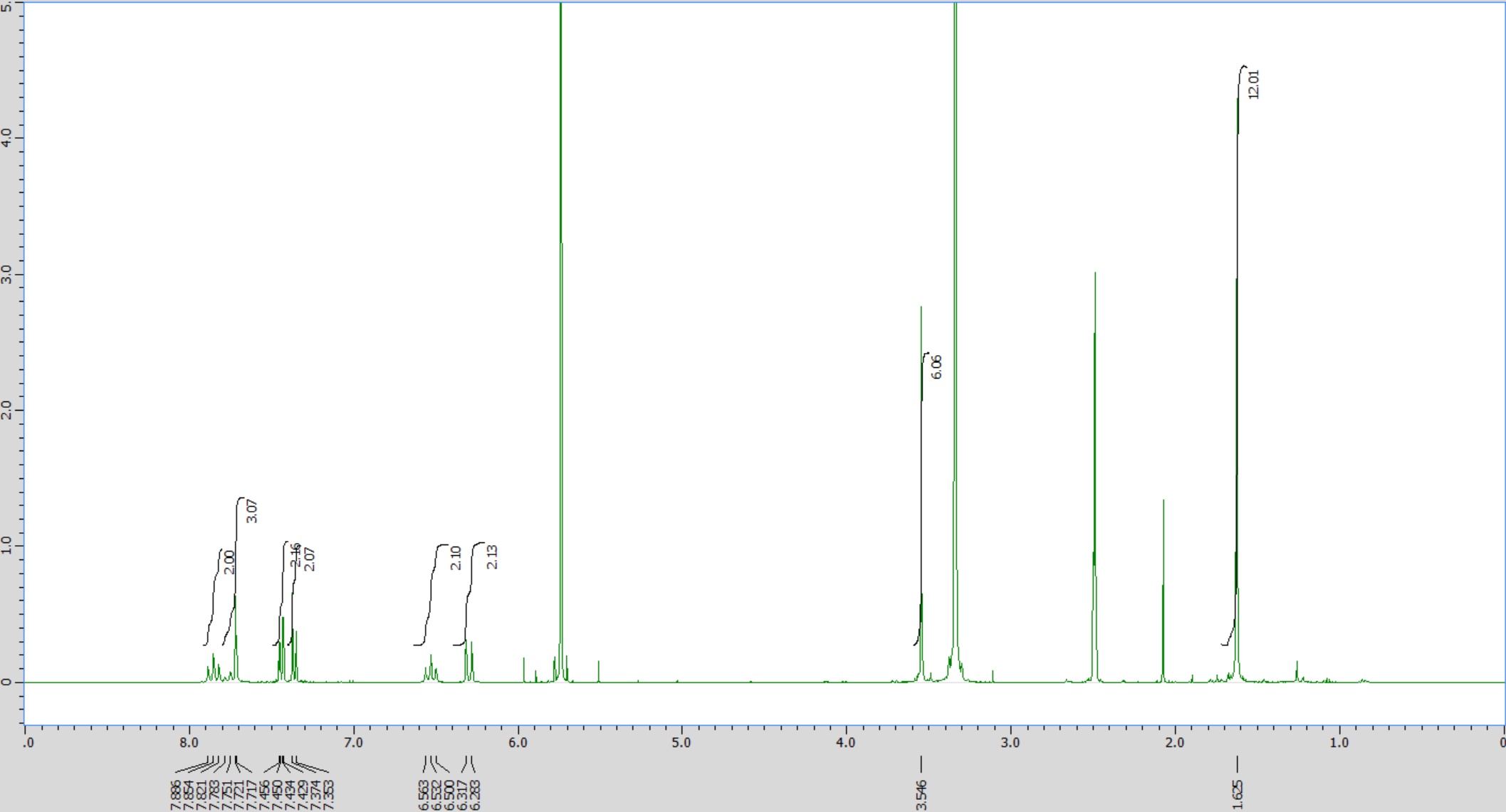

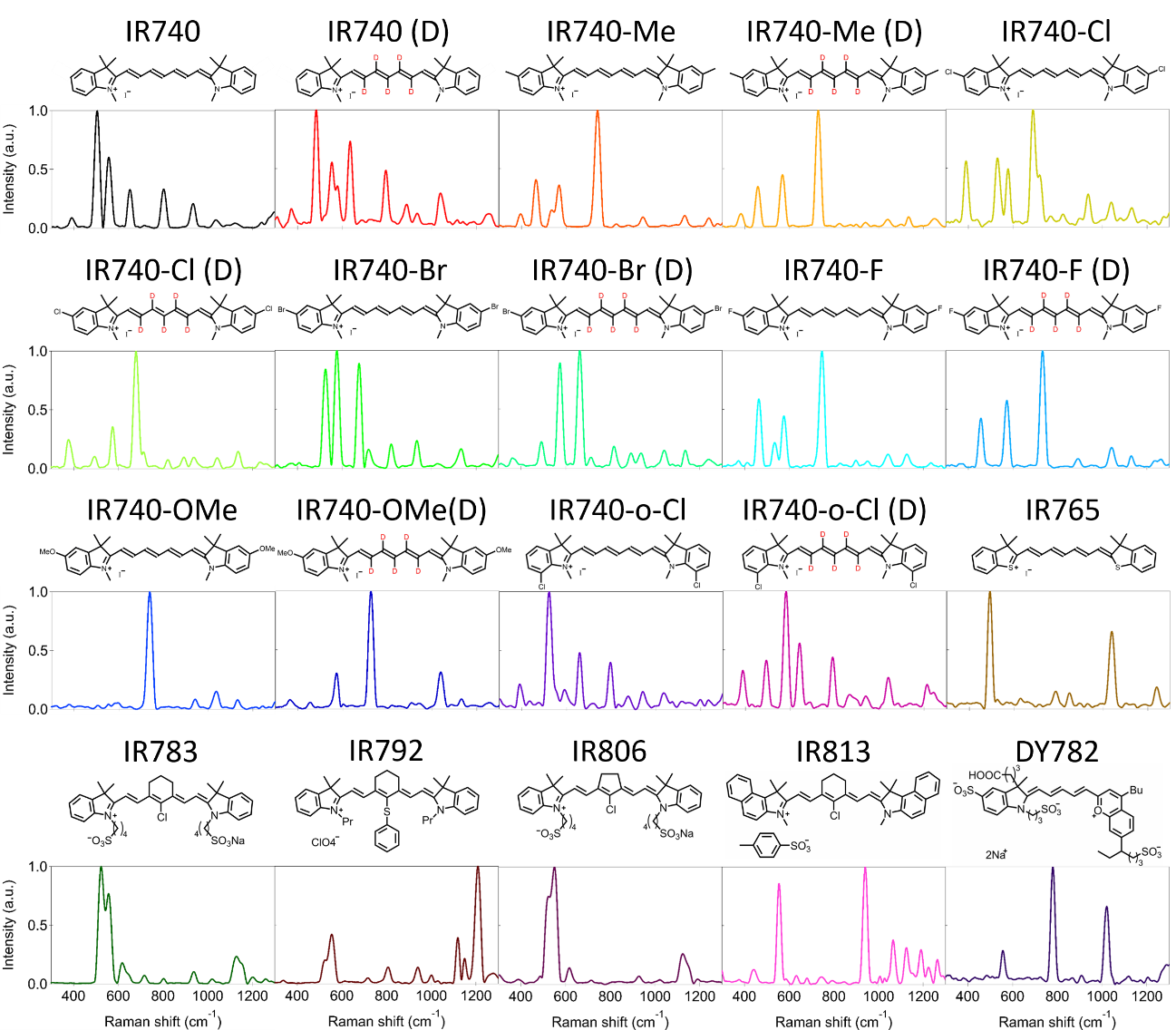
**

**Fig. S12. ^1^H NMR spectrum of IR740-Cl (DMSO-d6)**

**
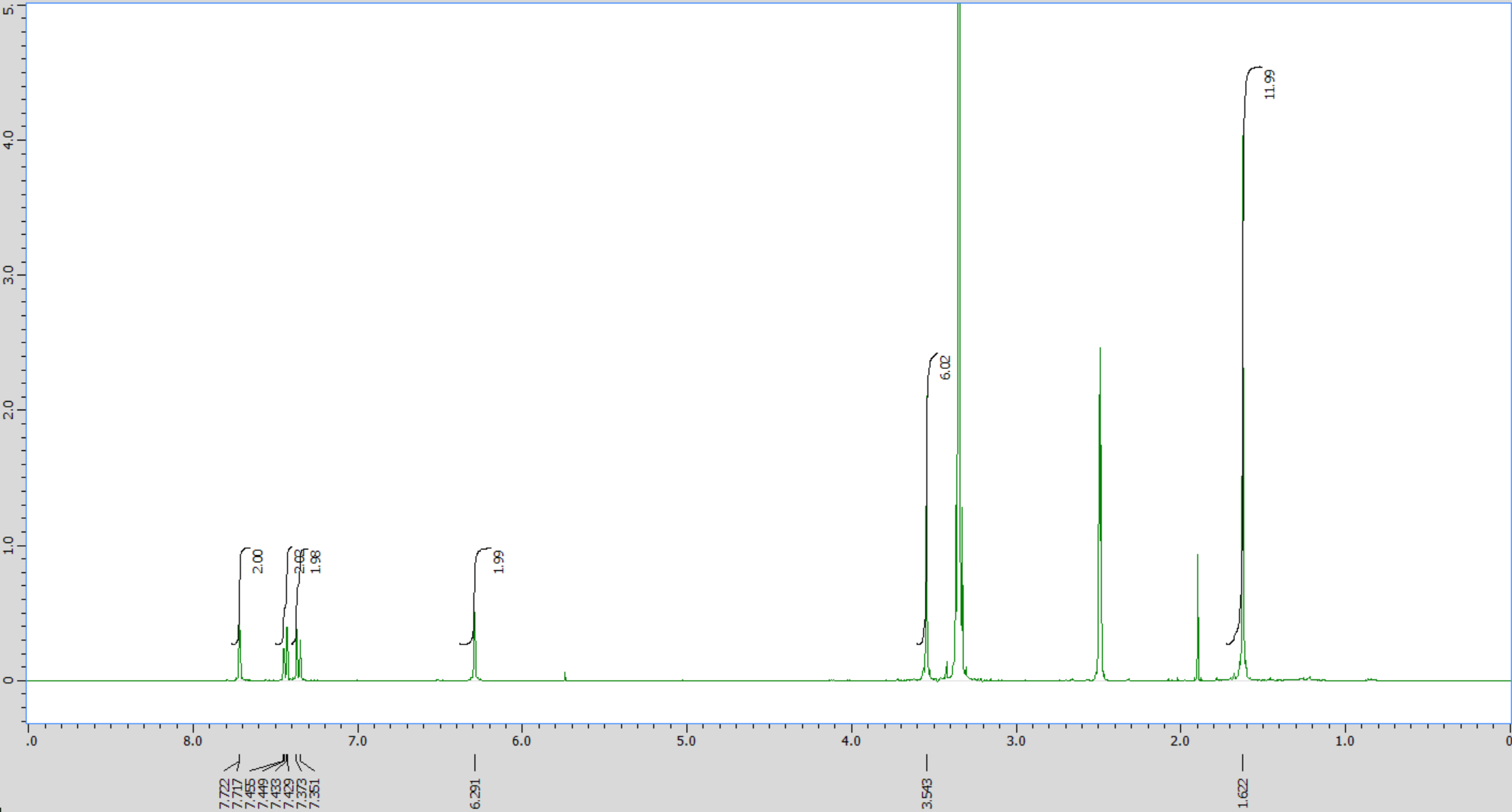

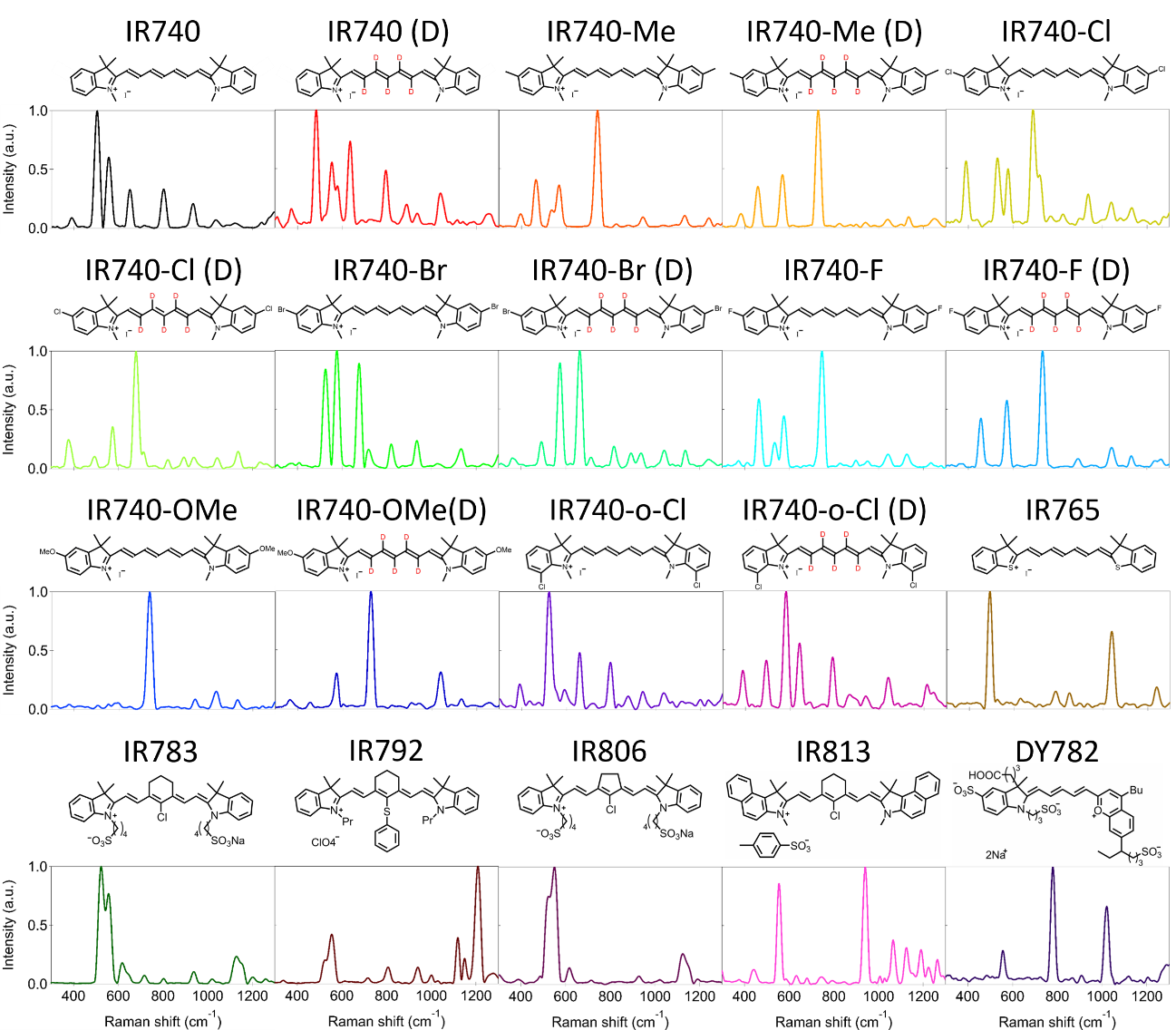
**

**Fig. S13. ^1^H NMR spectrum of IR740-Cl (D) (DMSO-d6)**

**
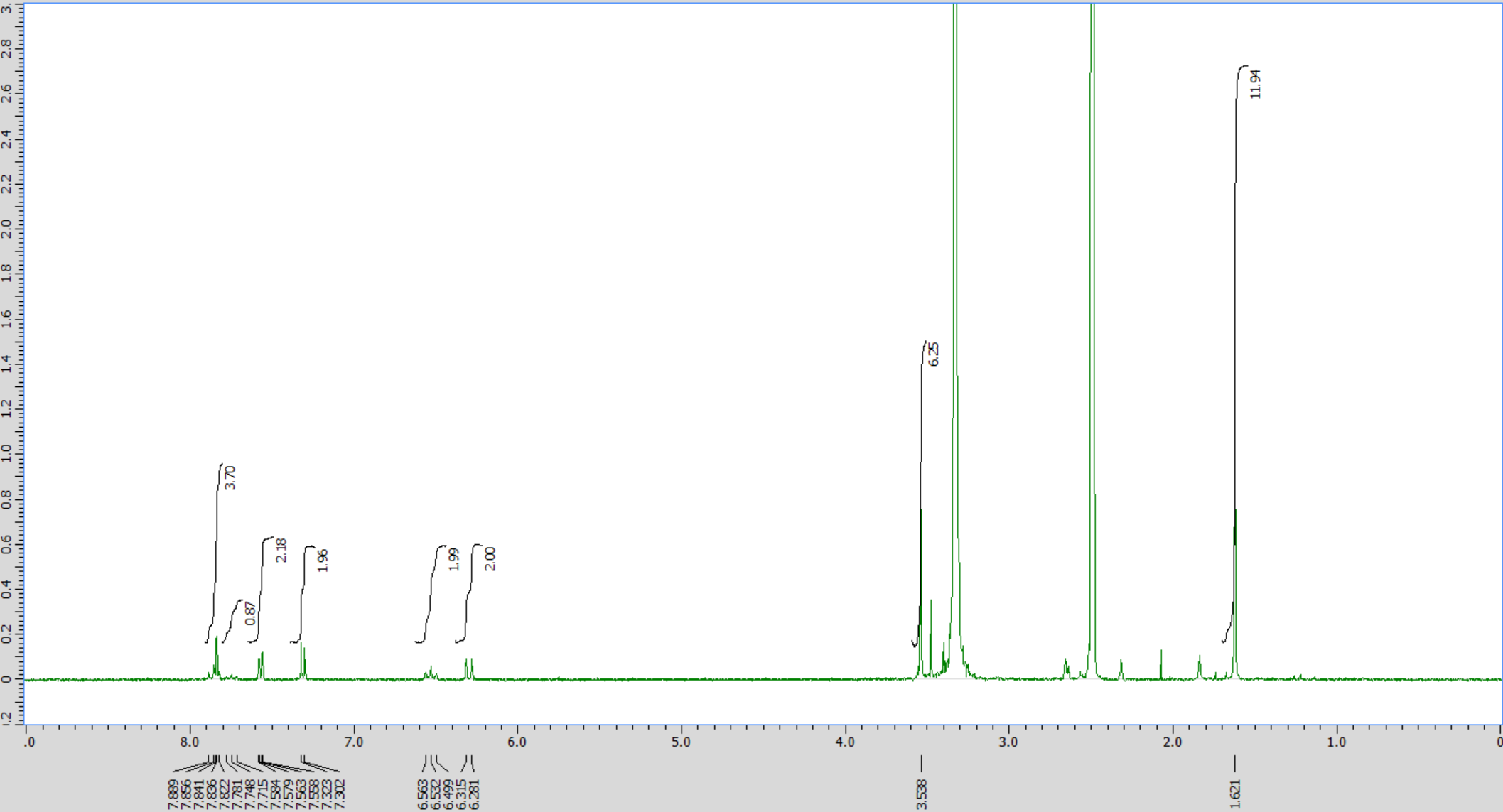

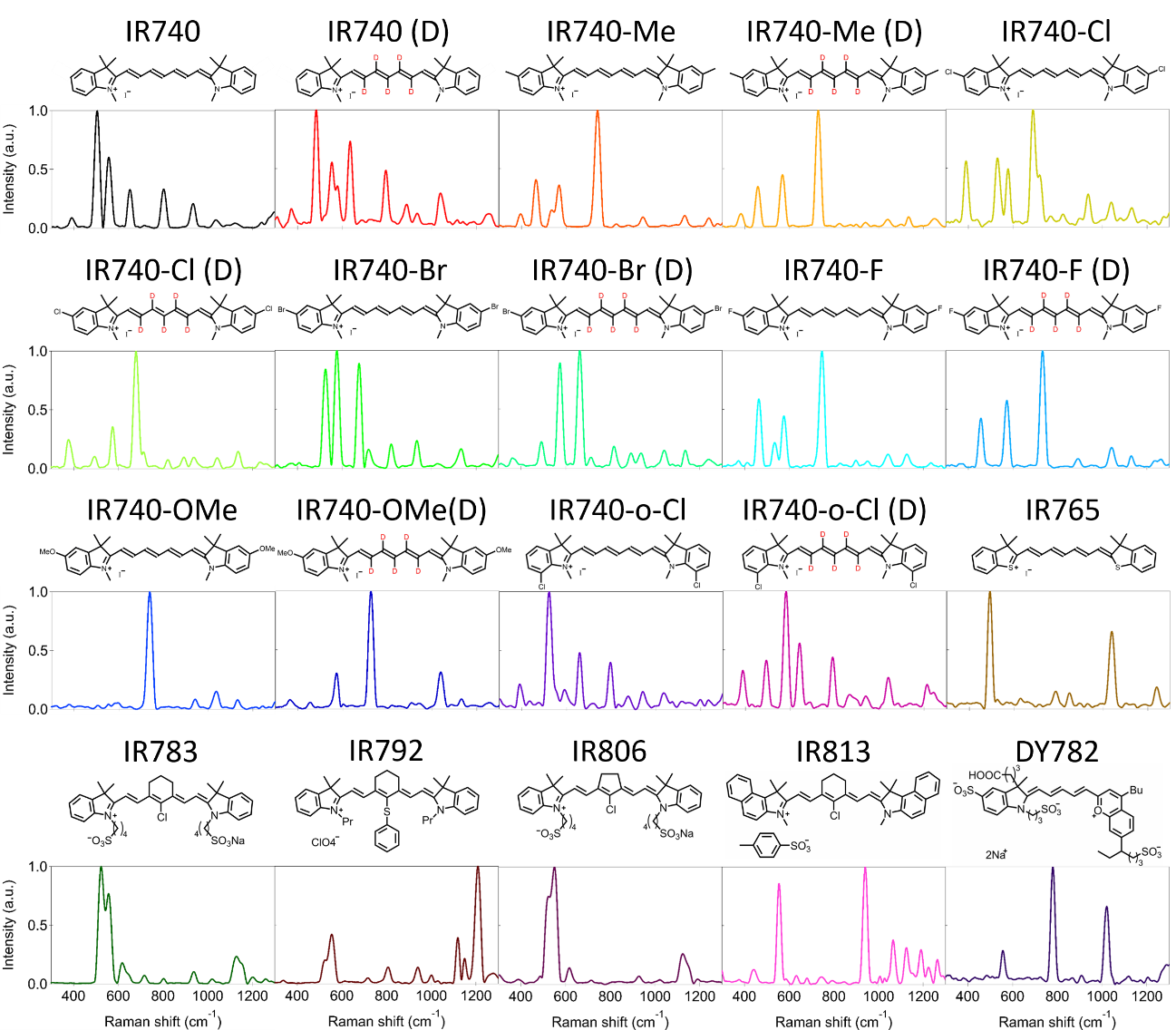
**

**Fig. S14. ^1^H NMR spectrum of IR740-Br (D) (DMSO-d6)**

**
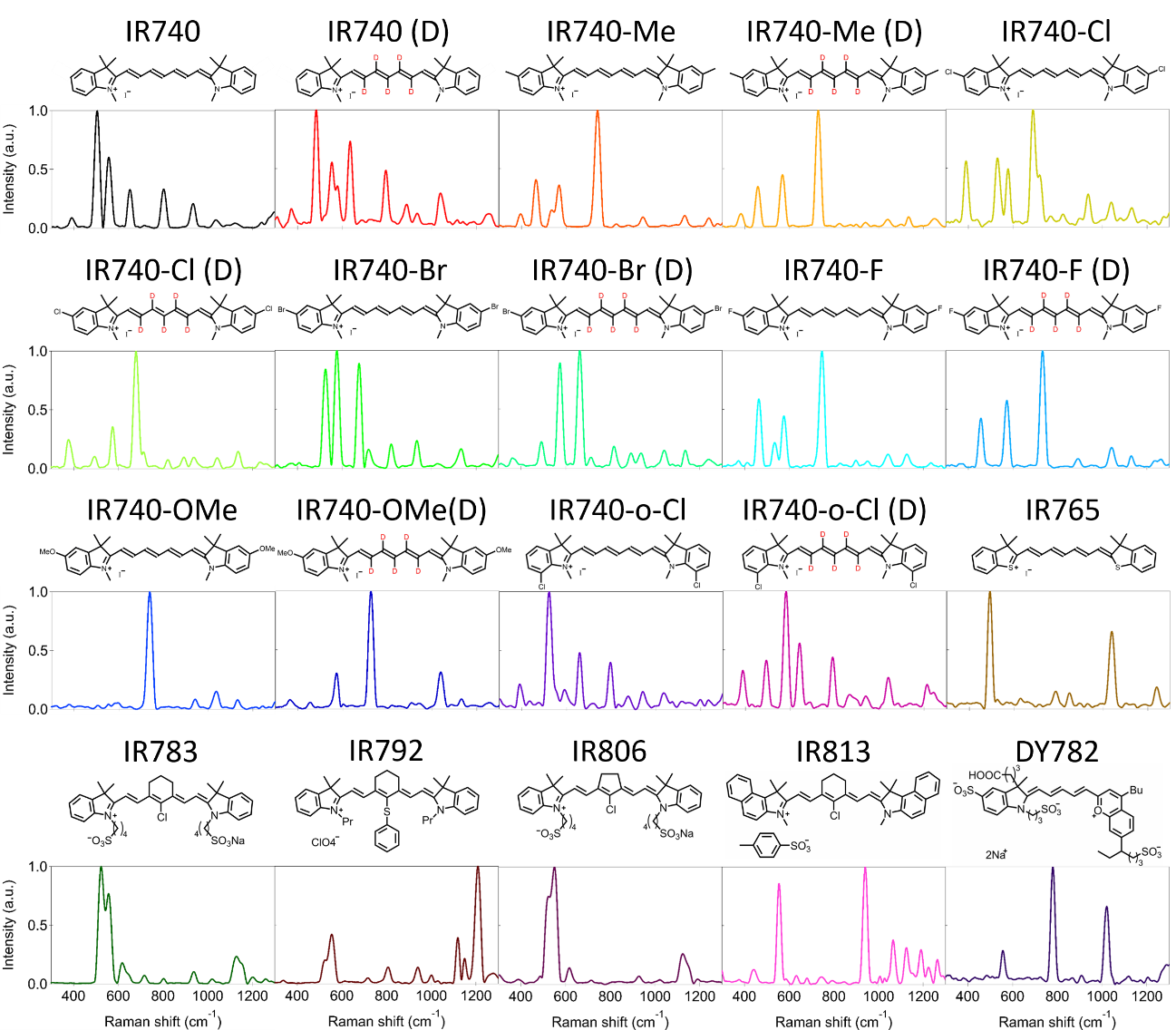

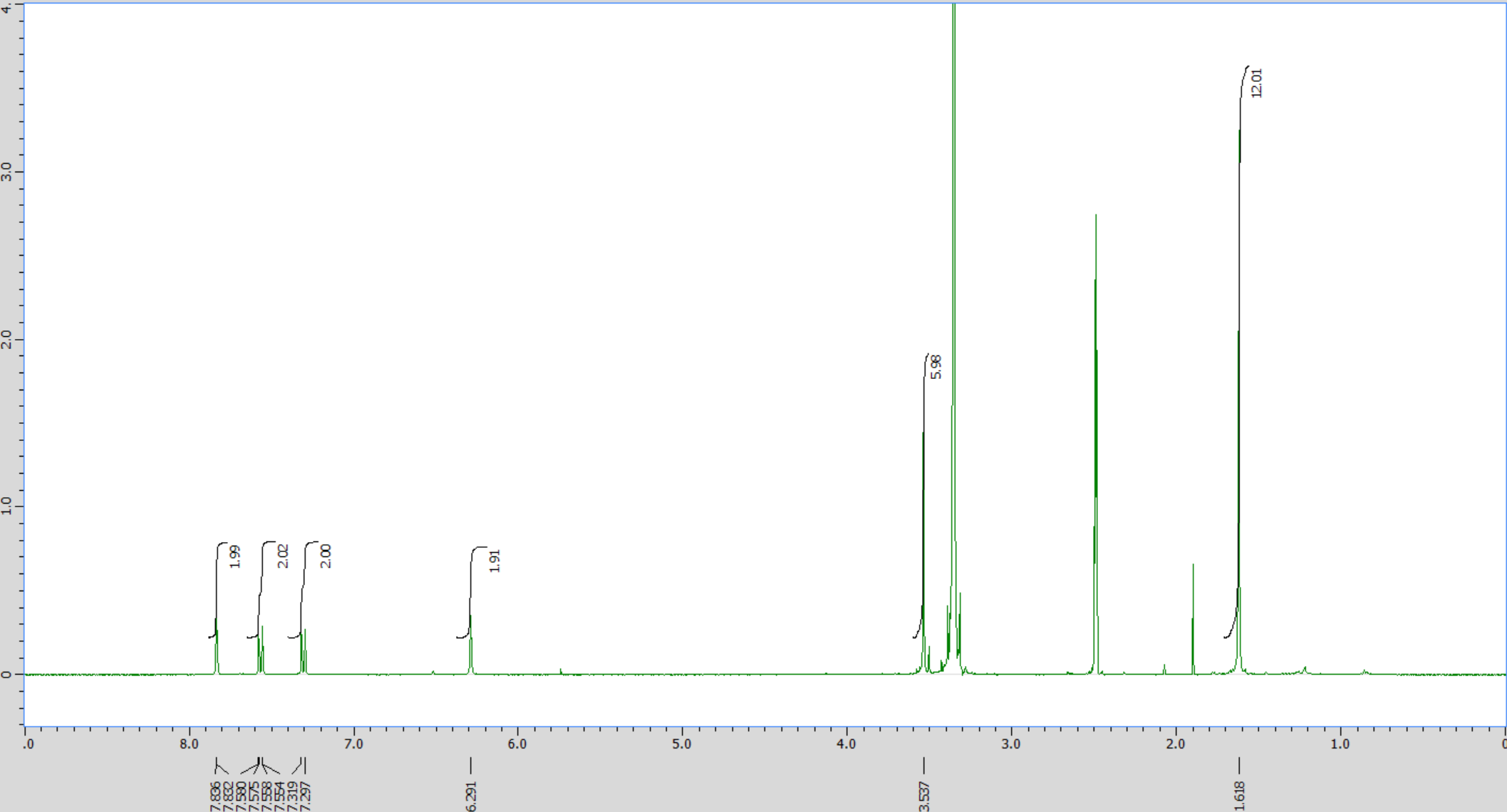
**

**Fig. S15. ^1^H NMR spectrum of IR740-Br (D) (DMSO-d6)**

**
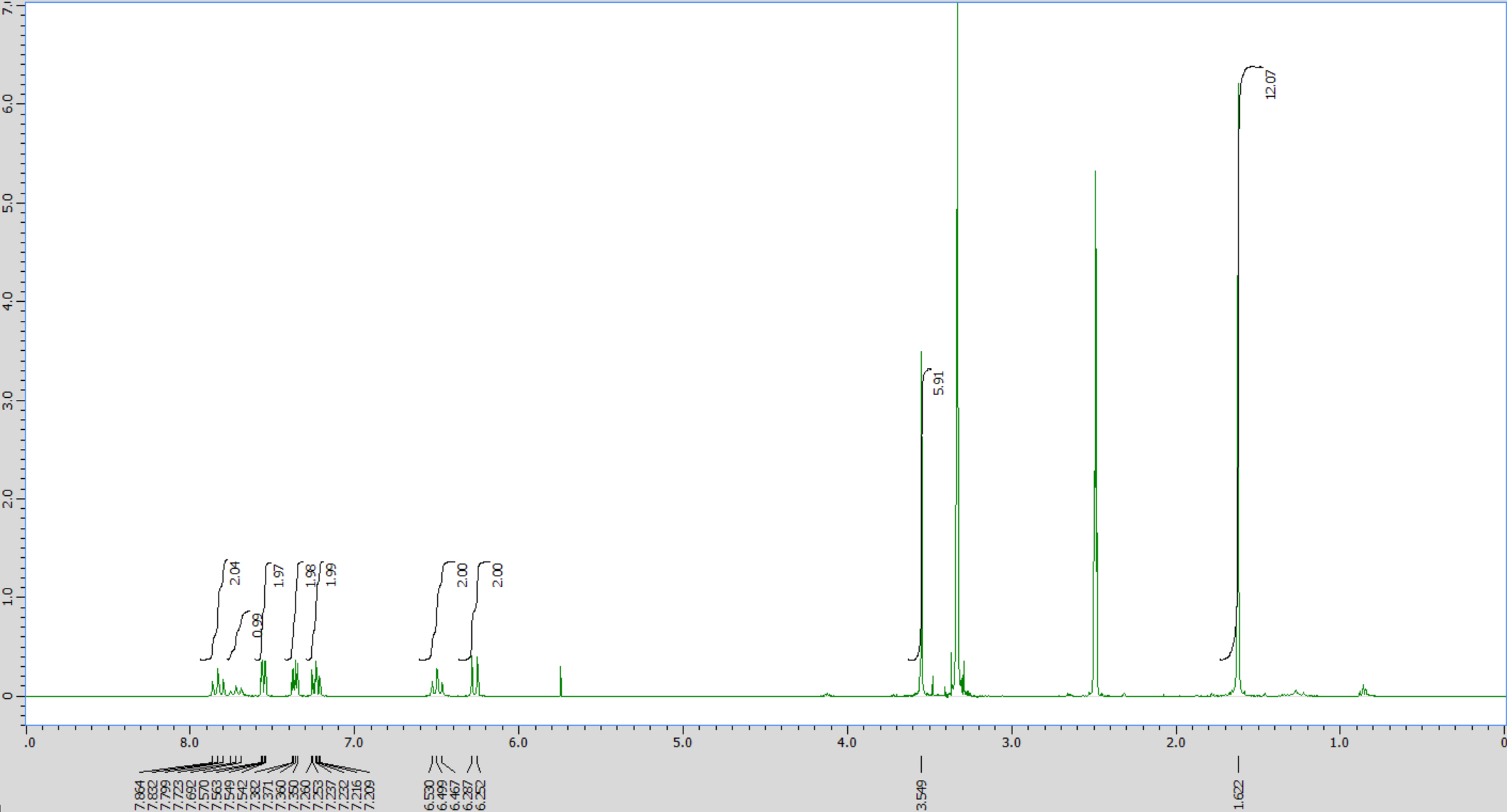

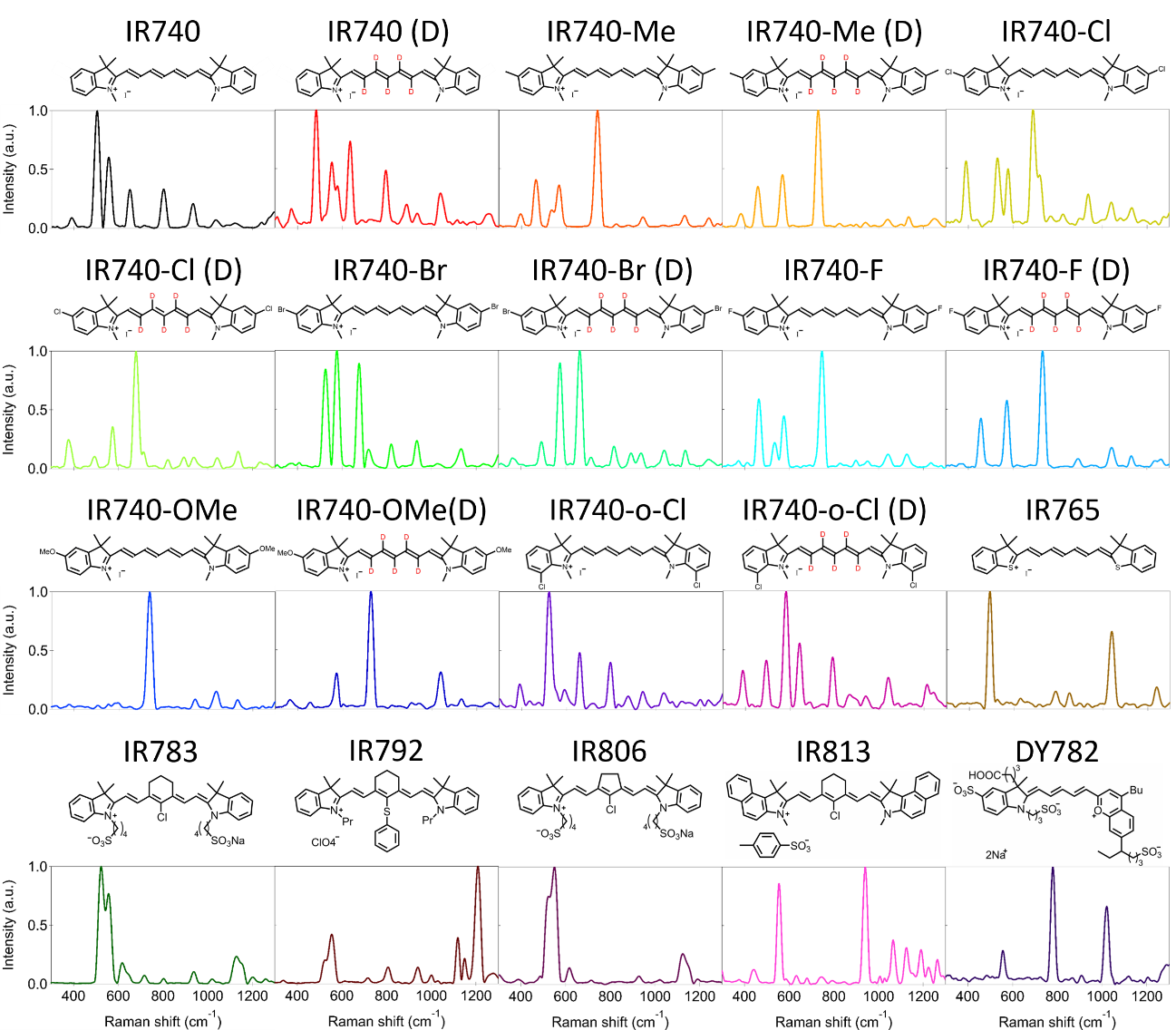
**

**Fig. S16. ^1^H NMR spectrum of IR740-F (DMSO-d6)**

**
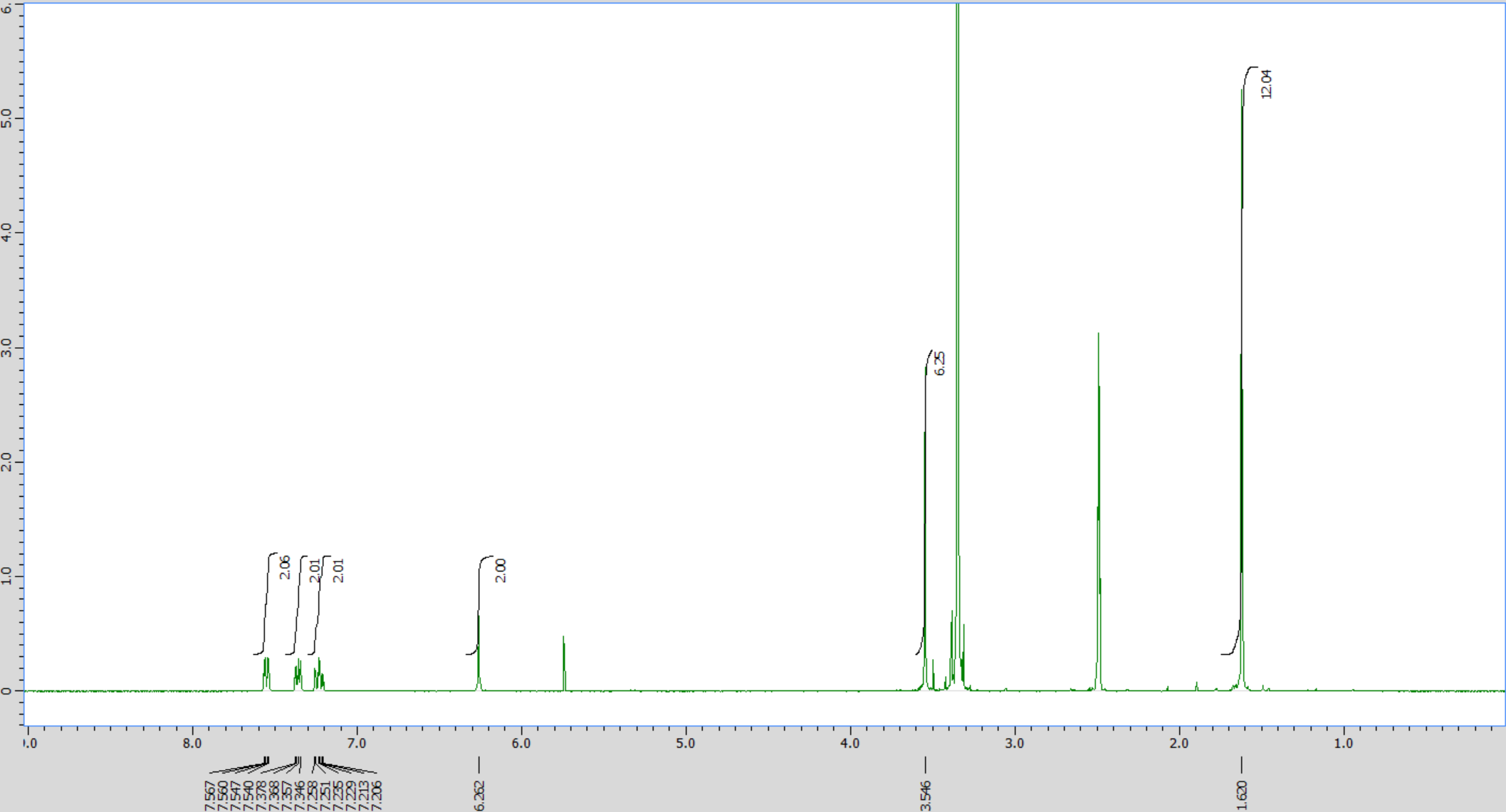

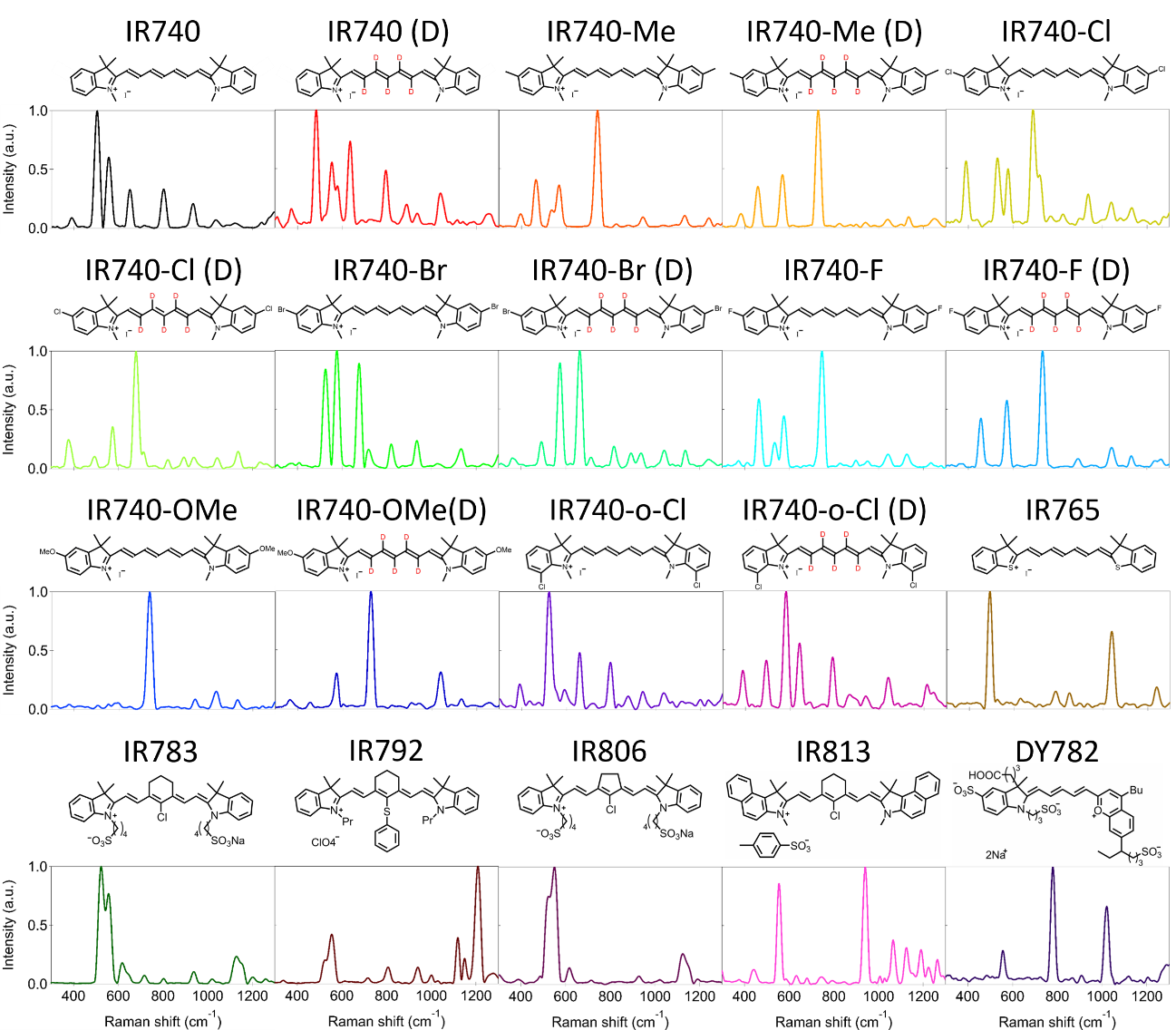
**

**Fig. S17. ^1^H NMR spectrum of IR740-F (D) (DMSO-d6)**

**
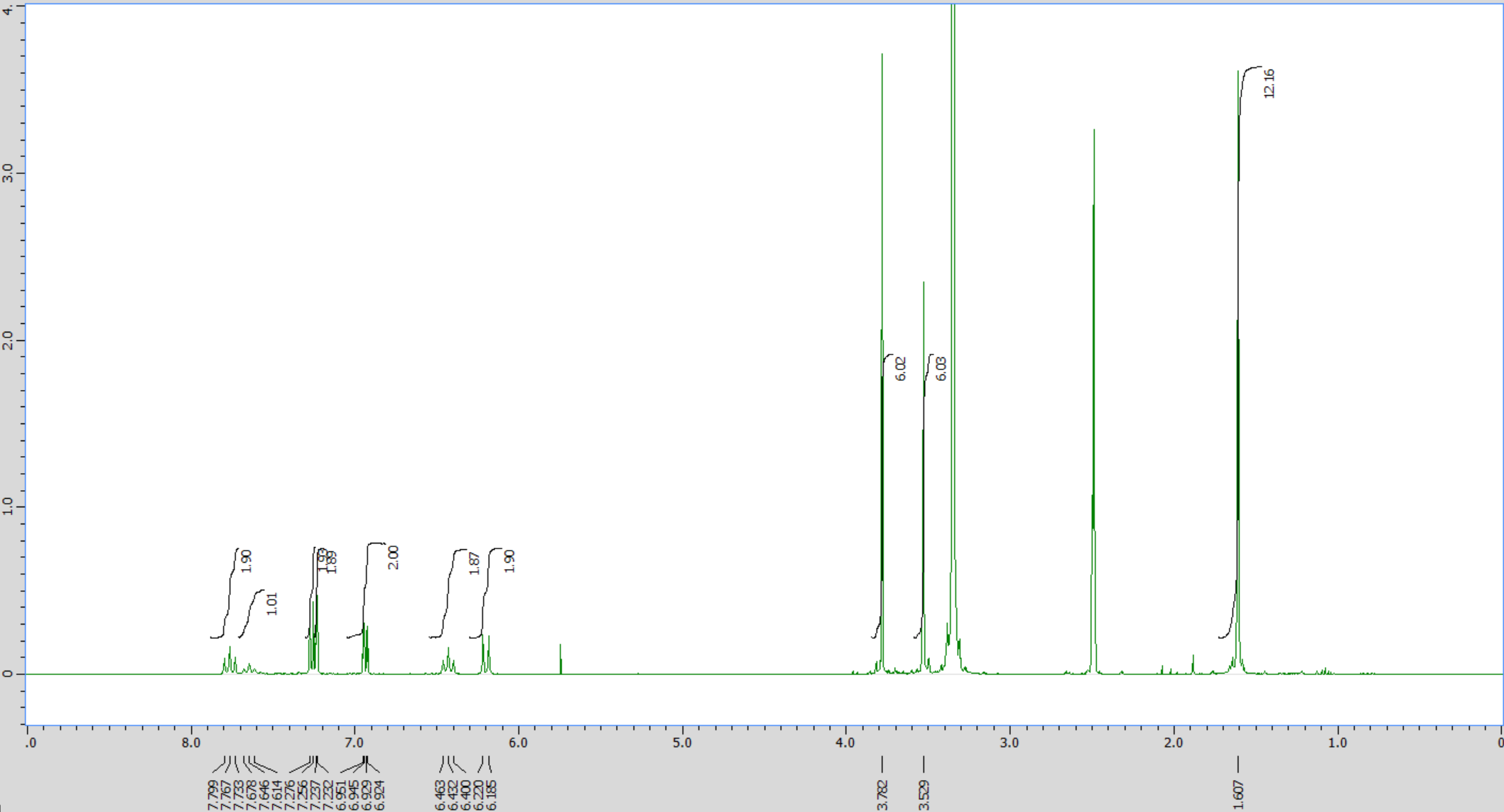

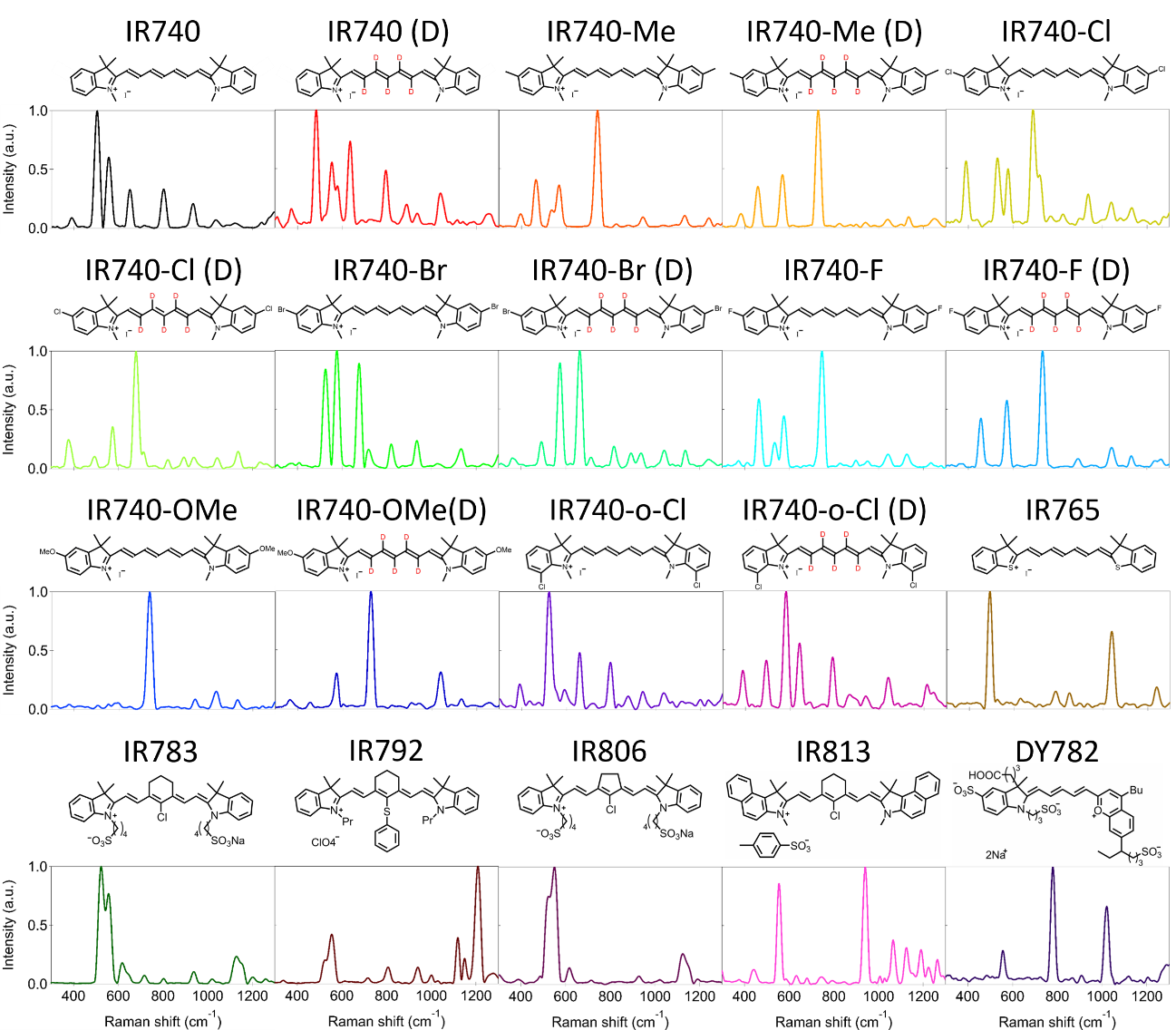
**

**Fig. S18. ^1^H NMR spectrum of IR740-OMe (DMSO-d6)**

**
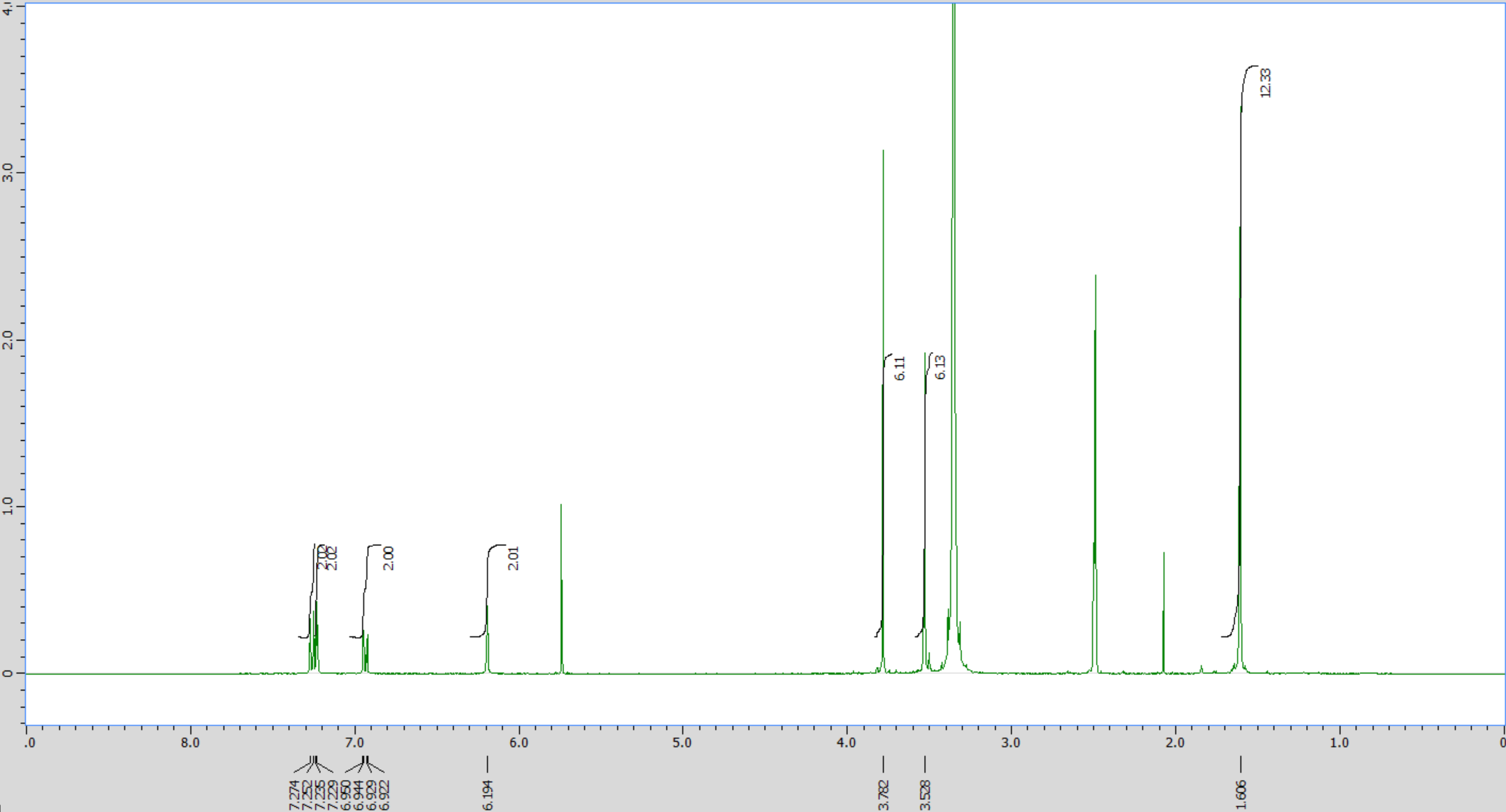

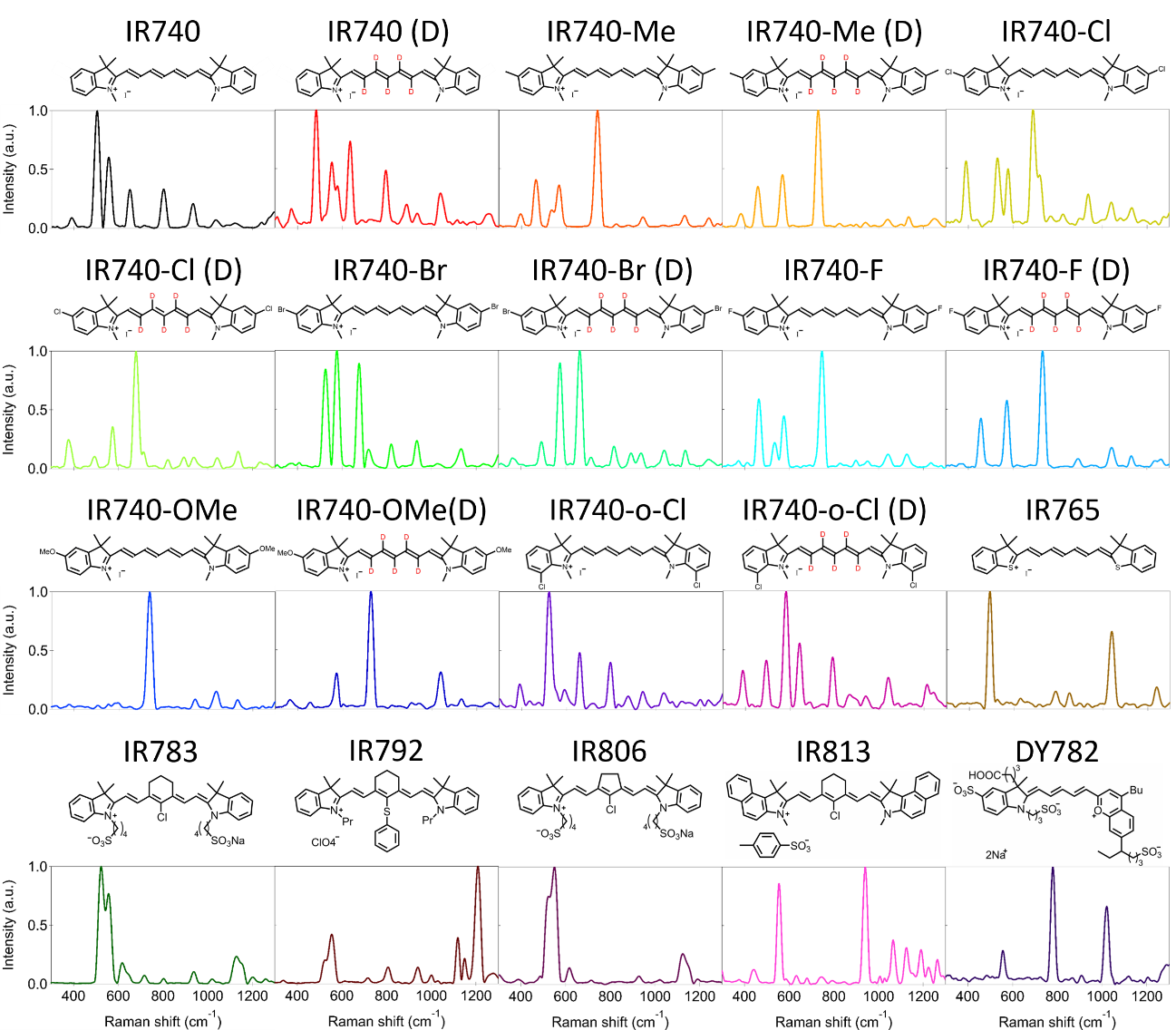
**

**Fig. S19. ^1^H NMR spectrum of IR740-OMe (D) (DMSO-d6)**

**
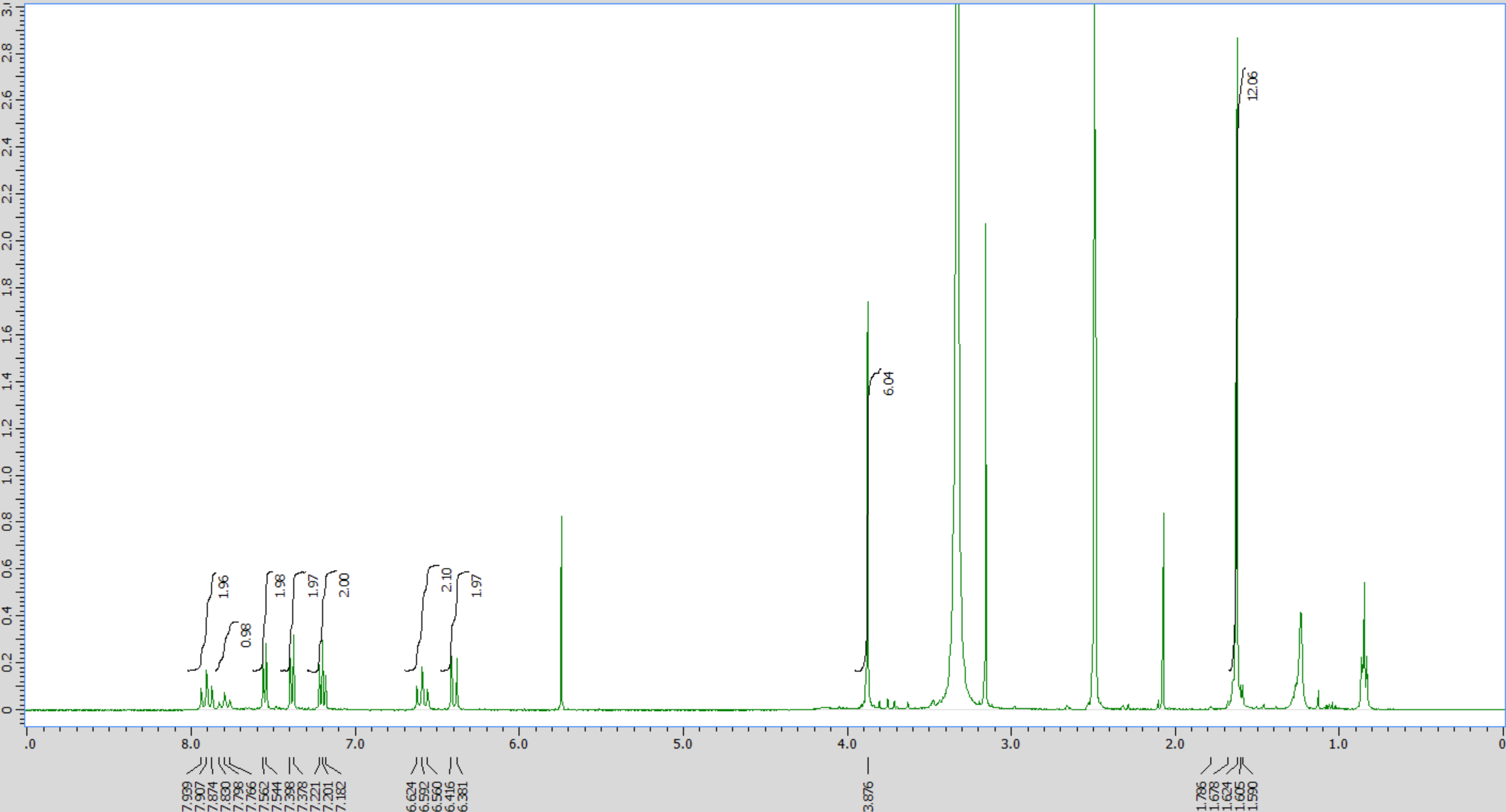

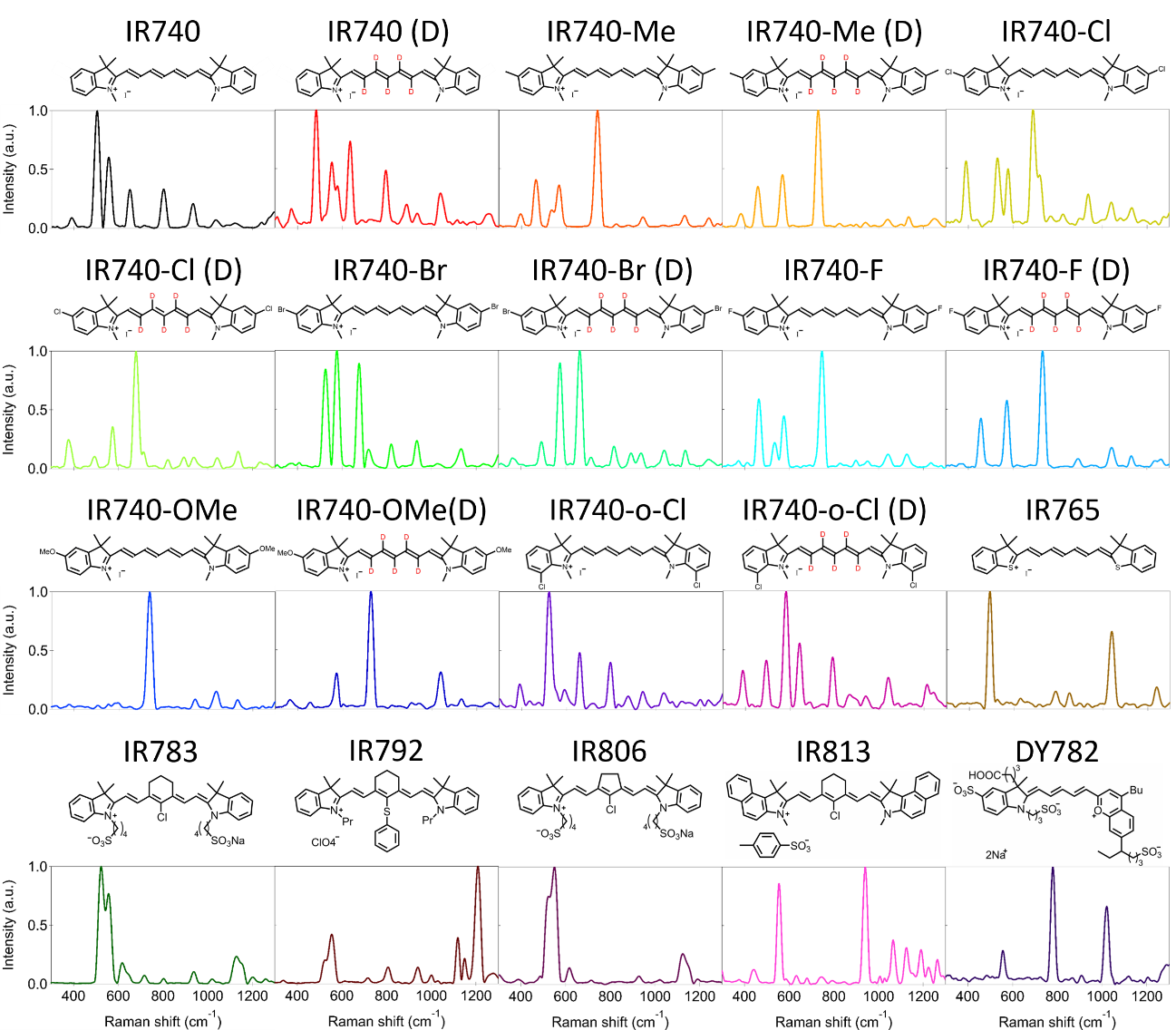
**

**Fig. S20. ^1^H NMR spectrum of IR740-o-Cl (DMSO-d6)**

**
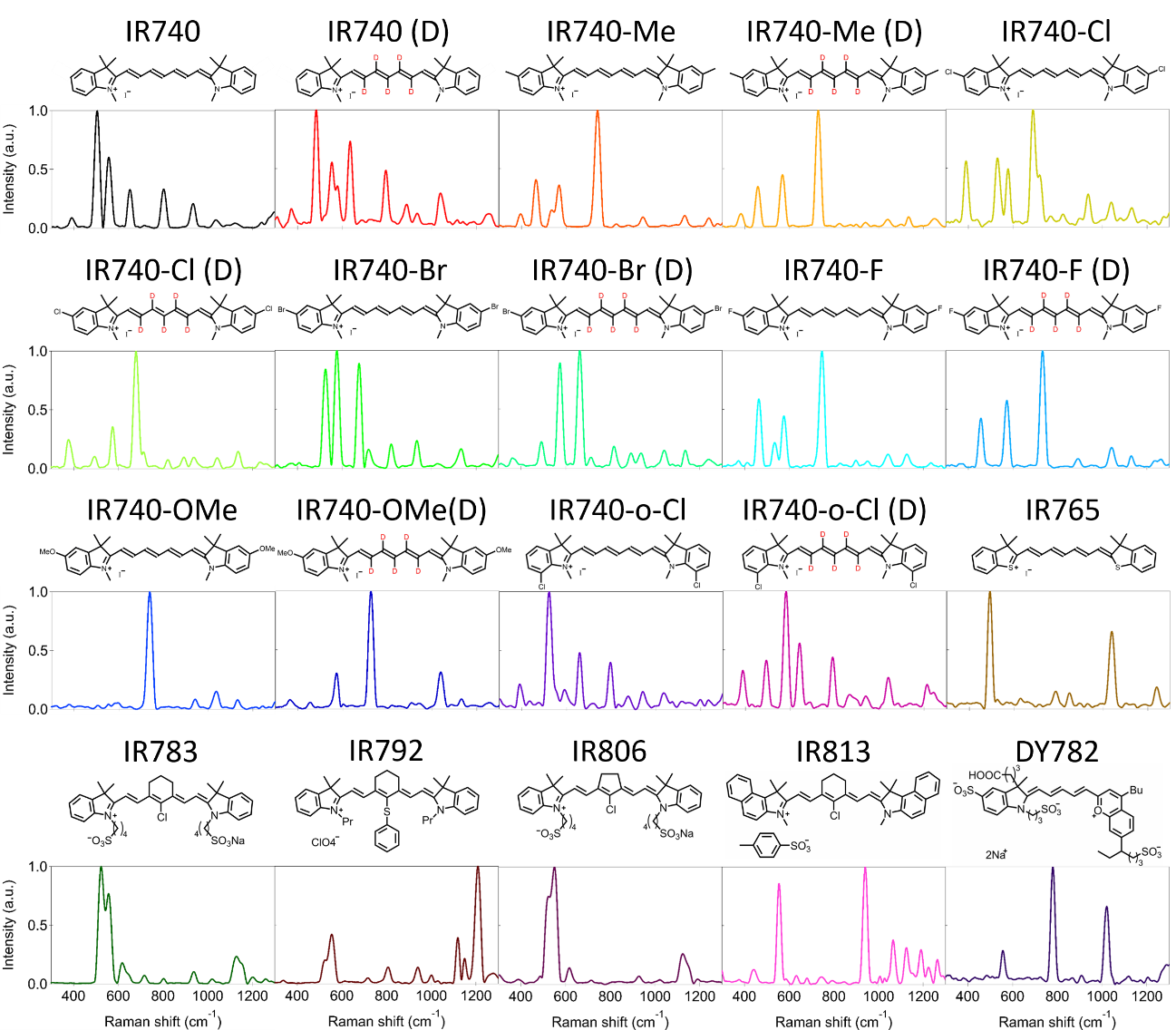

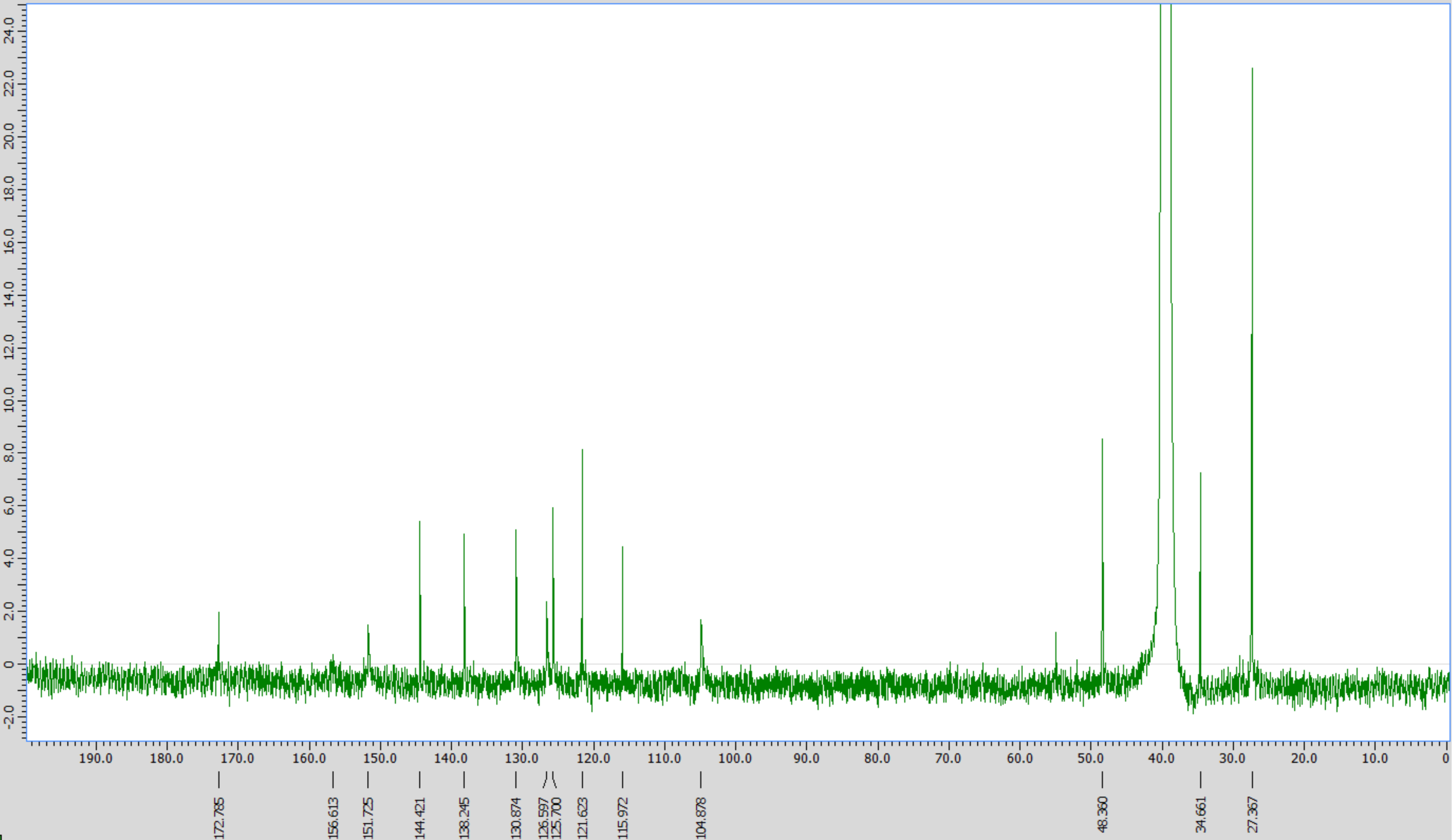
**

**Fig. S21. ^13^C NMR spectrum of IR740-o-Cl (DMSO-d6)**

**
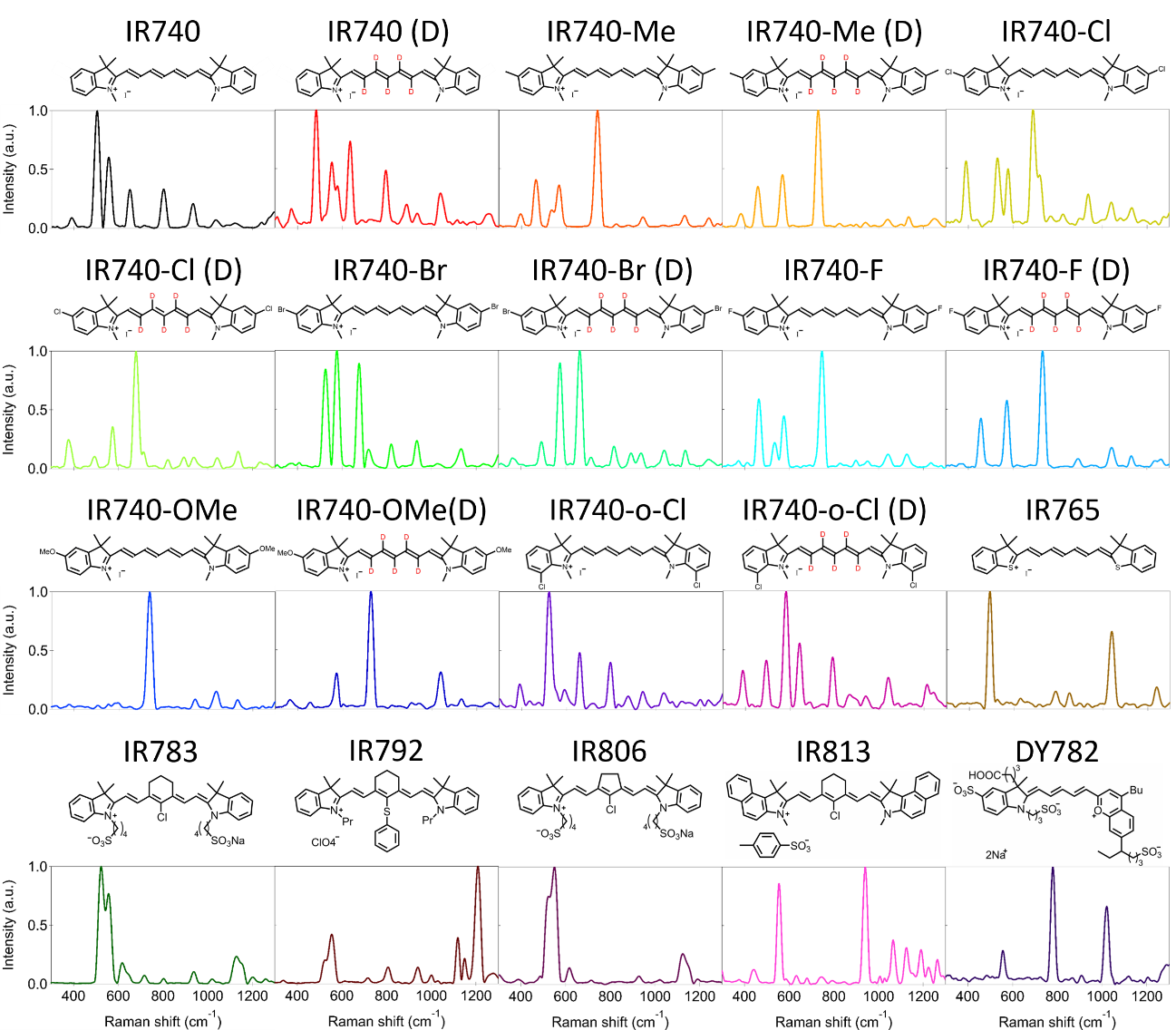
^
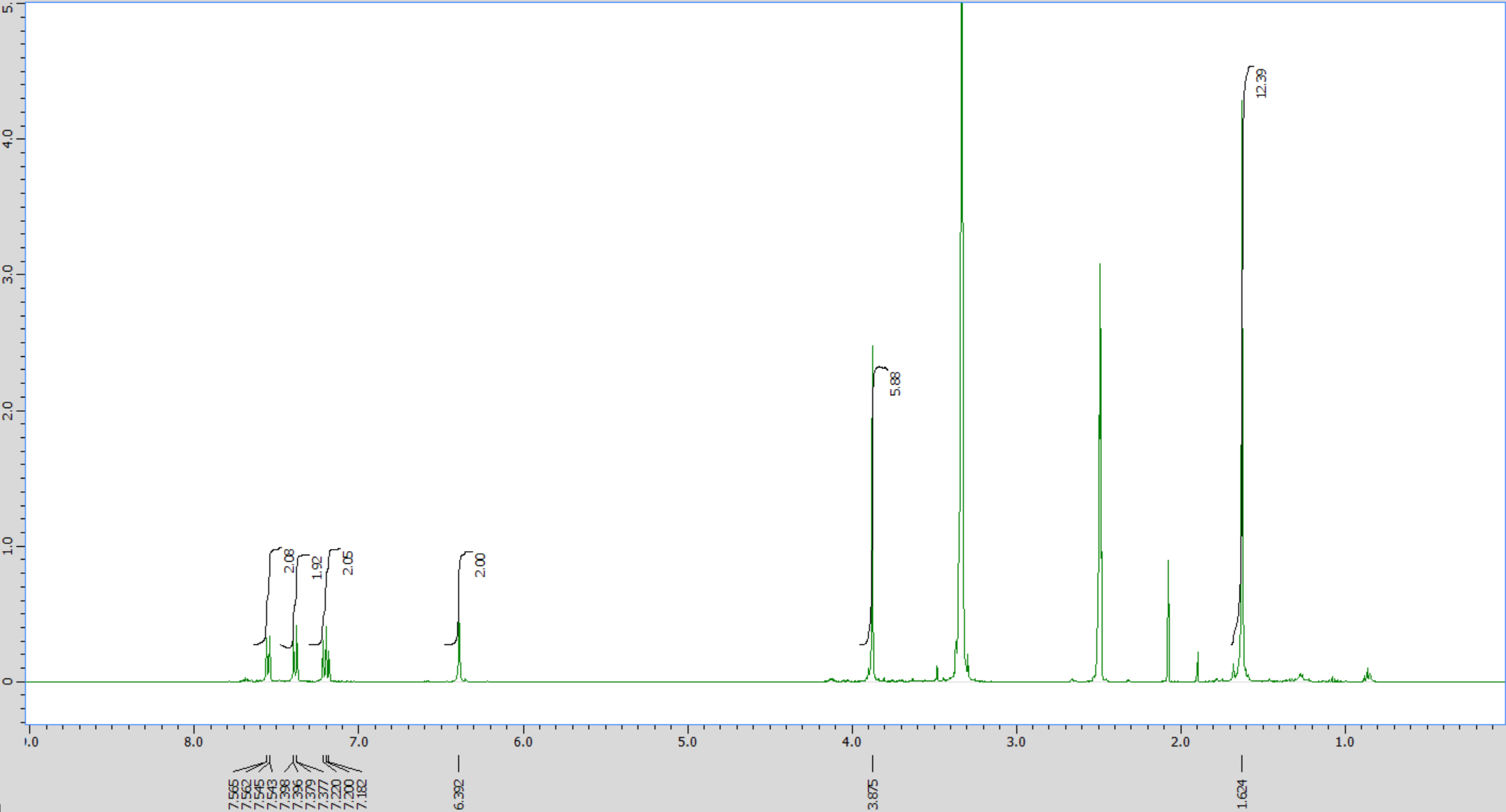
^**

**Fig. S22. ^1^H NMR spectrum of IR740-o-Cl (D) (DMSO-d6)**

**SI References**

1. A. Cossarizza, *et al.*, Guidelines for the use of flow cytometry and cell sorting in immunological studies (second edition). *European Journal of Immunology* **49** (2019).

2. N. Nitta, *et al.*, Intelligent Image-Activated Cell Sorting. *Cell* **175** (2018).

3. H. Mikami, *et al.*, Virtual-freezing fluorescence imaging flow cytometry. *Nature Communications* **11** (2020).

4. M. Brown, C. Wittwer, Flow cytometry: Principles and clinical applications in hematology. *Clinical Chemistry* **46**, 1221–1229 (2000).

5. M. Nishikawa, *et al.*, Massive image-based single-cell profiling reveals high levels of circulating platelet aggregates in patients with COVID-19. *Nature Communications* **12**, 1–12 (2021).

6. J. Brummelman, *et al.*, Development, application and computational analysis of high-dimensional fluorescent antibody panels for single-cell flow cytometry. *Nature Protocols* **14**, 1946–1969 (2019).

7. E. Lugli, M. Roederer, A. Cossarizza, Data analysis in flow cytometry: The future just started. *Cytometry Part A* **77** (2010).

8. K. M. Dean, A. E. Palmer, Advances in fluorescence labeling strategies for dynamic cellular imaging. *Nature Chemical Biology* **10** (2014).

9. T. Niehörster, *et al.*, Multi-target spectrally resolved fluorescence lifetime imaging microscopy. *Nature Methods* **13** (2016).

10. L. Wei, *et al.*, Super-multiplex vibrational imaging. *Nature* **544**, 465–470 (2017).

11. S. Schmutz, M. Valente, A. Cumano, S. Novault, Spectral cytometry has unique properties allowing multicolor analysis of cell suspensions isolated from solid tissues. *PLoS ONE* **11** (2016).

12. J. P. Nolan, D. Condello, Spectral Flow Cytometry. *Current Protocols in Cytometry* **63**, 1.27.1-1.27.13 (2013).

13. G. Grégori, *et al.*, Hyperspectral cytometry at the single-cell level using a 32-channel photodetector. *Cytometry Part A* **81 A**, 35–44 (2012).

14. L. M. Park, J. Lannigan, M. C. Jaimes, OMIP-069: Forty-Color Full Spectrum Flow Cytometry Panel for Deep Immunophenotyping of Major Cell Subsets in Human Peripheral Blood. *Cytometry Part A* **97**, 1044–1051 (2020).

15. S. C. Bendall, *et al.*, Single-cell mass cytometry of differential immune and drug responses across a human hematopoietic continuum. *Science* **332**, 687–696 (2011).

16. F. J. Hartmann, S. C. Bendall, Immune monitoring using mass cytometry and related high-dimensional imaging approaches. *Nature Reviews Rheumatology* **16**, 87–99 (2020).

17. F. Hu, *et al.*, Supermultiplexed optical imaging and barcoding with engineered polyynes. *Nature Methods* **15**, 194–200 (2018).

18. K. Hiramatsu, *et al.*, High-throughput label-free molecular fingerprinting flow cytometry. *Science Advances* **5**, 241–257 (2019).

19. J. Gala De Pablo, M. Lindley, K. Hiramatsu, K. Goda, High-Throughput Raman Flow Cytometry and beyond. *Accounts of Chemical Research* **54** (2021).

20. M. Lindley, J. Gala de Pablo, R. Kinegawa, K. Hiramatsu, K. Goda, Highly sensitive Fourier-transform coherent anti-Stokes Raman scattering spectroscopy via genetic algorithm pulse shaping. *Optics Letters* **46** (2021).

21. K. Hiramatsu, K. Yamada, M. Lindley, K. Suzuki, K. Goda, Large-scale label-free single-cell analysis of paramylon in Euglena gracilis by high-throughput broadband Raman flow cytometry. *Biomedical Optics Express* **11** (2020).

22. M. Cui, M. Joffre, J. Skodack, J. P. Ogilvie, “Interferometric Fourier transform coherent anti-stokes Raman scattering” (2006).

23. C. Chen, *et al.*, Multiplexed live-cell profiling with Raman probes. *Nature Communications* **12**, 1–13 (2021).

24. Z. Zhao, *et al.*, Ultra-bright Raman dots for multiplexed optical imaging. *Nature Communications* **12** (2021).

25. K. Dodo, *et al.*, Synthesis of deuterated γ-linolenic acid and application for biological studies: Metabolic tuning and Raman imaging. *Chemical Communications* **57**, 2180–2183 (2021).

26. S. Egoshi, K. Dodo, K. Ohgane, M. Sodeoka, Deuteration of terminal alkynes realizes simultaneous live cell Raman imaging of similar alkyne-tagged biomolecules. *Organic and Biomolecular Chemistry* **19**, 8232–8236 (2021).

27. H. Midi, A. Bagheri, Robust multicollinearity diagnostic measure in collinear data set in *International Conference on Applied Mathematics, Simulation, Modelling - Proceedings*, (2010).

28. J. H. Lee, I. J. Gomez, V. B. Sitterle, J. C. Meredith, Dye-labeled polystyrene latex microspheres prepared via a combined swelling-diffusion technique. *Journal of Colloid and Interface Science* **363**, 137–144 (2011).

29. L. Van Der Maaten, G. Hinton, Visualizing data using t-SNE. *Journal of Machine Learning Research* **9** (2008).

30. D. M. Monti, *et al.*, Biocompatibility, uptake and endocytosis pathways of polystyrene nanoparticles in primary human renal epithelial cells. *Journal of Biotechnology* **193**, 3–10 (2015).

31. S. H. Hansen, K. Sandvig, B. Van Deurs, Clathrin and HA2 adaptors: Effects of potassium depletion, hypertonic medium, and cytosol acidification. *Journal of Cell Biology* **121** (1993).

32. J. Chen, *et al.*, Ultrabright Fluorescent Organic Nanoparticles Based on Small-Molecule Ionic Isolation Lattices**. *Angewandte Chemie - International Edition* **60** (2021).

33. E. A. Owens, *et al.*, Near-Infrared Illumination of Native Tissues for Image-Guided Surgery. *Journal of Medicinal Chemistry* **59**, 5311–5323 (2016).

34. H. Hyun, *et al.*, Structure-inherent targeting of near-infrared fluorophores for parathyroid and thyroid gland imaging. *Nature Medicine* **21**, 192–197 (2015).

35. A. Böhm, T. Bach, Radical Reactions Induced by Visible Light in Dichloromethane Solutions of Hünig’s Base: Synthetic Applications and Mechanistic Observations. *Chemistry - A European Journal* **22**, 15921–15928 (2016).

36. E. N. Marvell, T. H. Li, C. Paik, Formation of phenylpyridinium chloride from 5-anilino-N-phenyl-2,4-pentadienylideniminium chloride in acidic media. *Tetrahedron Letters* **14**, 2089–2092 (1973).
